# Phase-pure 2D tin halide perovskite thin flakes for stable lasing

**DOI:** 10.1126/sciadv.adh0517

**Published:** 2023-08-09

**Authors:** Yahui Li, Hongzhi Zhou, Ming Xia, Hongzhi Shen, Tianyu Wang, Haikuo Gao, Xin Sheng, Yanxin Han, Zhong Chen, Letian Dou, Haiming Zhu, Enzheng Shi

**Affiliations:** ^1^ Research Center for Industries of the Future and School of Engineering, Westlake University, Hangzhou 310030, China.; ^2^ Hangzhou Global Scientific and Technological Innovation Center, Zhejiang University, Hangzhou 311215, China.; ^3^ Shandong Engineering Research Center of Aeronautical Materials and Devices, College of Aeronautical Engineering, Binzhou University, Binzhou 251900, China.; ^4^ Instrumentation and Service Center for Molecular Sciences, Westlake University, Hangzhou 310030, China.; ^5^ Davidson School of Chemical Engineering, Purdue University, West Lafayette, IN 47907, USA.

## Abstract

Ruddlesden-Popper tin halide perovskites are a class of two-dimensional (2D) semiconductors with exceptional optoelectronic properties, high carrier mobility, and low toxicity. However, the synthesis of phase-pure 2D tin perovskites is still challenging, and the fundamental understanding of their optoelectronic properties is deficient compared to their lead counterparts. Here, we report the synthesis of a series of 2D tin perovskite bulk crystals with high phase purity via a mixed-solvent strategy. By engineering the quantum-well thickness (related to *n* value) and organic ligands, the optoelectronic properties, including photoluminescence emission, exciton-phonon coupling strength, and exciton binding energy, exhibit a wide tunability. In addition, these 2D tin perovskites exhibited excellent lasing performance. Both high–*n* value tin perovskite (*n* > 1) and *n* = 1 tin perovskite thin flakes were successfully optically pumped to lase. Furthermore, the lasing from 2D tin perovskites could be maintained up to room temperature. Our findings highlight the tremendous potential of 2D tin perovskites as promising candidates for high-performance lasers.

## Teaser

Phase-pure 2D tin perovskite crystals demonstrate exceptional lasing performance.

## INTRODUCTION

Van der Waals (vdW) semiconductors, such as transition metal dichalcogenides (TMDCs) and black phosphorus, have been widely exploited in optics, electronics, and optoelectronics (*1*–*6*). Their ease of mechanical exfoliation into thin flakes ensures the ultraflat surface free of dangling bonds and the precise control of thickness in subnanometer scale (*7*). As an emerging class of vdW semiconductors with high luminescence yield and widely tunable band structures, two-dimensional (2D) Ruddlesden-Popper (RP) halide perovskites L_2_A_*n*−1_M*_n_*X_3*n*+1_ (L: large organic cations, A: small organic cations, M: divalent metal cations, X: halide, and *n*: metal halide octahedral number in each layer of RP perovskite along the out-of-plane direction) have gained tremendous attention and rapid development (*8*–*12*). Unlike TMDCs, 2D halide perovskites are organic-inorganic hybrid materials. The semiconductor properties of 2D halide perovskites are primarily dominated by the inorganic metal halide octahedral layers, and a relatively large distance is formed between adjacent inorganic layers by the organic spacer ligands, reducing the interlayer coupling, and preserving the direct bandgap property in perovskites of arbitrary thickness (*10*). Moreover, the organic ligands act as molecular-level encapsulation layers, mitigating the interaction between the polar octahedra and ambient moisture/oxygen, thus leading to improved environmental stability over their 3D counterparts (*13*, *14*). Currently, 2D lead halide perovskites are commonly used as active or passivation layers in optoelectronic devices (*15*–*19*), with 2D perovskite–based lasers gradually gaining attention. For instance, a series of mechanically exfoliated 2D lead perovskites (*n* > 1) have been found to exhibit homologous lasing without external cavities in the visible region, such as (BA)_2_MA_*n*−1_Pb*_n_*I_3*n*+1_ (BA^+^: butylammonium, MA: methylammonium) (*19*). However, lasing in 2D lead halide perovskite (*n* = 1) without external cavities is difficult to achieve and was only reported in bulk crystals (*20*) or thin films with the help of external cavities (*21*, *22*), which was assumed to be the result of high auger recombination rate and strong exciton-phonon coupling strength (*19*). On the other hand, tin-based alternatives exhibit smaller optical bandgaps and smaller effective masses for carriers and are less toxic compared to lead-based perovskites (*23*–*25*). Unfortunately, limited by the poor stability and fast crystal nucleation/growth rate, the synthesis of phase-pure RP tin halide perovskites is still challenging, and their corresponding optoelectronic properties are lack of investigation.

Here, a series of highly phase-pure 2D RP tin perovskite bulk crystals, including (BA)_2_MA_*n*−1_Sn*_n_*I_3*n*+1_ (*n* = 1 to 4), (PEA)_2_MA_*n*−1_Sn*_n_*I_3*n*+1_ (PEA^+^: phenethylammonium and *n* = 1, 2), (2T)_2_MA_*n*−1_Sn*_n_*I_3*n*+1_ (*n* = 1 and 2) (2T^+^: bithiophenylethylammonium), and (3T)_2_MA_*n*−1_Sn*_n_*I_3*n*+1_ (*n* = 1 and 2) {3T^+^: 2-([2,2′:5′,2″-terthiophen]-5-yl)ethan- 1-aminium}, were successfully synthesized via a new crystallization method featuring a mixed solvent with controlled polarity. The exciton-phonon coupling strength and effective exciton binding energy of these 2D tin perovskites were extracted from their temperature-dependent photoluminescence (PL) spectra or absorption spectra. Mechanically exfoliated thin flakes from these bulk crystals demonstrate exceptional lasing performance, among which 2D tin perovskites (*n* > 1) with BA^+^ ligand exhibited tunable lasing emission from red to near-infrared wavelength. In addition, optically pumped lasing was achieved in 2D *n* = 1 tin perovskite by adopting conjugated ligands instead of BA^+^. The lasing from 2D tin perovskite thin flakes demonstrated excellent stability and could be maintained even at room temperature (300 K), which is a substantial advantage over their lead-based counterparts.

## RESULTS

The synthesis of high-purity 2D tin halide perovskite single crystals remains a great challenge due to the easy oxidation of divalent tin to tetravalent tin. By precisely tuning the ratio of different precursors, the polarity of growth solvent, and controlling the growth conditions with minimized exposure to ambient air (see Materials and Methods and table S1), highly phase-pure 2D tin halide perovskite single crystals were synthesized. For perovskites with BA^+^ or PEA^+^ as spacer ligands, as they can be easily dissolved in aqueous solvent system composed of HI and H_3_PO_2_, the corresponding bulk crystals (BA)_2_MA_*n*−1_Sn*_n_*I_3*n*+1_ (*n* = 1 to 4, abbreviated as BA Sn *n* = 1, BA Sn *n* = 2, BA Sn *n* = 3, and BA Sn *n* = 4, respectively) and (PEA)_2_MA_*n*−1_Sn*_n_*I_3*n*+1_ (*n* = 1 and 2, abbreviated as PEA Sn *n* = 1 and PEA Sn *n* = 2, respectively) were synthesized simply via the optimization of the precursor ratio.

**Fig. 1.**
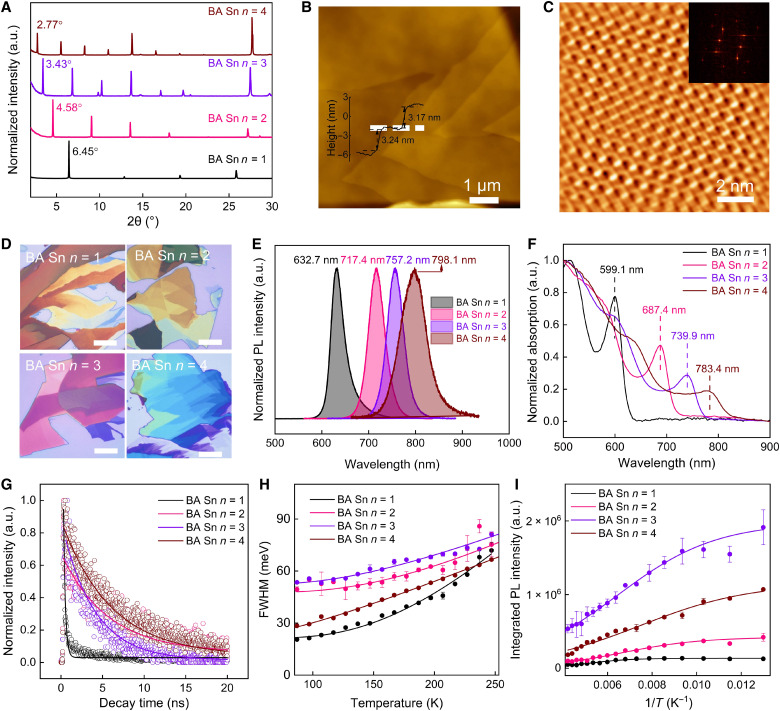
Characterizations of (BA)_2_MA_*n*−1_Sn*_n_*I_3*n*+1_ thin flakes. (**A**) PXRD patterns of (BA)_2_MA_*n*−1_Sn*_n_*I_3*n*+1_ (*n* = 1 to 4). a.u., arbitrary units. (**B**) AFM image of exfoliated (BA)_2_MA_3_Sn_4_I_13_ thin flake. (**C**) Fourier filtered high-resolution AFM image of exfoliated (BA)_2_MA_3_Sn_4_I_13_ thin flake. (**D**) Bright-field optical images of (BA)_2_MA_*n*−1_Sn*_n_*I_3*n*+1_ (*n* = 1 to 4). Scale bar, 10 μm. (**E**) PL spectra of (BA)_2_MA_*n*−1_Sn*_n_*I_3*n*+1_ (*n* = 1 to 4). (**F**) UV-vis absorption spectra of (BA)_2_MA_*n*−1_Sn*_n_*I_3*n*+1_ (*n* = 1 to 4). (**G**) TRPL measurements of (BA)_2_MA_*n*−1_Sn*_n_*I_3*n*+1_ (*n* = 1 to 4). (**H**) Extraction of exciton-phonon coupling strength by fitting the FWHM versus temperature. (**I**) Exciton binding energies obtained from Arrhenius formula fitting.

The phase purity of as-synthesized (BA)_2_MA_*n*−1_Sn*_n_*I_3*n*+1_ (*n* = 1 to 4) crystals was confirmed by powder x-ray diffraction (PXRD), from which no impurity signals were observed (Fig. 1A). The high phase purity also avoids carrier transfer between perovskites with different *n* values and guarantees the pure color of luminescence. The thickness of monolayer perovskite (*n* = 1 to 4) can be determined by probing the terraces in exfoliated 2D perovskite flakes using atomic force microscope (AFM) characterization. For example, the monolayer thickness of (BA)_2_MA_*n*−1_Sn*_n_*I_3*n*+1_ is 1.37 nm (BA Sn *n* = 1), 1.93 nm (BA Sn *n* = 2), 2.57 nm (BA Sn *n* = 3), and 3.18 nm (BA Sn *n* = 4) (Fig. 1B and figs. S1 and S2), which is consistent with the values calculated from PXRD profiles and single-crystal structure (Fig. 1A and tables S2 to S5). To further determine the sample quality, the surface state was directly resolved by using lateral force microscopy (LFM) operating in contact mode with the tip scanning orthogonal to the long axis of the cantilever. As shown in Fig. 1C and fig. S3, subnanometer lattice fringes on the sample surface were clearly seen. The organic ligands are uniformly arranged without observable micro pinholes or defects from the high-resolution image after 2D fast Fourier transform. This further indicates that the exfoliated perovskite thin flakes are of highly ordered surface. In addition, optical and PL characterizations were conducted to reveal the relationship between the structure and optical properties of these tin halide perovskites with tunable *n* values. These vdW materials have a black appearance, and the corresponding exfoliated thin flakes generally have arbitrary thickness and shape (Fig. 1D and figs. S4 and S5). With the *n* value increasing from 1 to 4, the PL emission peak increases from 632 to ~800 nm (Fig. 1E). From the ultraviolet-visible (UV-vis) absorption spectra (Fig. 1F), the position of the room-temperature lowest-energy excitonic absorption peak increases from 599.1 to 783.4 nm accordingly, and its intensity relative to the continuum absorption decreases due to the decreasing exciton binding energy with the *n* value increasing from 1 to 4. At the same time, the Stokes shift, defined as the difference between the excitonic absorption peak and the excitonic PL peak, decreases from 34 to 15 nm (Fig. 1, E and F), indicating that the high *n* value (*n* > 1) perovskite has smaller energy loss than (BA)_2_SnI_4_. Time-resolved PL (TRPL) measurements were conducted to study the exciton dynamic behaviors of exfoliated (BA)_2_MA_*n*−1_Sn*_n_*I_3*n*+1_ crystals. It is shown that (BA)_2_MA_*n*−1_Sn*_n_*I_3*n*+1_ (*n* = 2 to 4) perovskites have longer radiative recombination lifetime than (BA)_2_SnI_4_ perovskites. From the TRPL measurements, the carrier lifetime is 0.22 ns for (BA)_2_SnI_4_, 5.59 ns for (BA)_2_MASn_2_I_7_, 3.93 ns for (BA)_2_MA_2_Sn_3_I_10_, and 5.90 ns for (BA)_2_MA_3_Sn_4_I_13_, which is likely caused by the gradual weakening of quantum confinement effect as the *n* value increases (Fig. 1G and fig. S6).

The performance of optoelectronic devices of halide perovskites is usually intimately associated with the exciton-phonon coupling strength, which limits the carrier mobility and represents how carriers interact with the phonons. We performed temperature- dependent PL measurements of (BA)_2_MA_*n*−1_Sn*_n_*I_3*n*+1_ (*n* = 1 to 4; fig. S7). On the basis of the first-order perturbation, the temperature-dependent PL linewidth broadening can be caused by three scattering mechanisms (*26*–*28*). According to previous studies of RP halide perovskites, considering the Fröhlich interaction between charge carriers and longitudinal optical (LO) phonons is very strong in ionic compounds (*29*), LO phonons are generally the dominant phonons in the full width at half maximum (FWHM) broadening, and the contribution from acoustic phonons and impurity scattering can be neglected (*27*, *30*). In view of this, the correlation between the FWHM of (BA)_2_MA_*n*−1_Sn*_n_*I_3*n*+1_ and temperature was fitted using the LO phonon dominating model, as shown below

$$\text{Γ}(T)={\text{Γ}}_{0}+\frac{{\text{Γ}}_{\mathrm{LO}}}{\left(e^{E_{\mathrm{LO}}/k_{B}T-1}\right)}$$ (1)

In the above formula, Γ_0_ is a nonuniform broadening term independent of temperature, Γ_LO_ is the coupling strength between exciton and LO phonons, *E*_LO_ is an energy representative of the frequency for the LO phonon branch, *T* is the temperature, and *k*_B_ is the Boltzmann constant. From fig. S7, we can find that the PL peaks of (BA)_2_MA_*n*−1_Sn*_n_*I_3*n*+1_ (*n* = 2 to 4) blue-shifted gradually during the cooling process from 247 to 77 K. However, the PL peak sharply red-shifted to ~578 nm at 257 K for (BA)_2_SnI_4_, which is the indication of structural phase transition (*31*). After fitting the temperature-dependent full width at half maximum (FWHW) of PL spectra according to Eq. 1, the coupling strength Γ_LO_ is calculated to be 547 meV for (BA)_2_SnI_4_ (corresponding to the phase of <257 K), 228 meV for (BA)_2_MASn_2_I_7_, 136 meV for (BA)_2_MA_2_Sn_3_I_10_, and 62 meV for (BA)_2_MA_3_Sn_4_I_13_ (Fig. 1H, fig. S7, and table S6). This result indicates that the interaction between the excitons and phonons can be effectively suppressed, and the energy loss can be reduced via increasing the *n* value. It is important to highlight that the crystallinity of the perovskite and the density of point defects can influence Γ_LO_. Therefore, it is expected that there are variations in Γ_LO_ when compared to those reported in other literatures. For example, for spin-coated (PEA)_2_SnI_4_ thin film, the measured Γ_LO_ is 199.3 meV by Zhang *et al.* (*31*) and 14 meV by Hansen *et al.* (*32*), indicating that the difference in material preparation method would lead to the discrepancy in exciton-phonon coupling strength of 2D tin halide perovskites. Besides, the effective exciton binding energy of the perovskite can be extracted by using the Arrhenius formula fitting (*32*–*34*)

$$I(T)=\frac{I_{0}}{1+Ae^{\left(-E_{b}/k_{B}T\right)}}$$ (2)

where *I*(*T*) is the integrated PL intensity, *I*_0_ is the integrated PL intensity at 0 K, *A* is a constant, and *E*_b_ is the effective exciton binding energy. From this formula, the calculated exciton binding energies are 93, 49, 39, and 35 meV corresponding to (BA)_2_SnI_4_, (BA)_2_MASn_2_I_7_, (BA)_2_MA_2_Sn_3_I_10_, and (BA)_2_MA_3_Sn_4_I_13_, respectively (Fig. 1I and table S6). Note that there exists discrepancy between the real *E*_b_ and the *E*_b_ extracted from the Arrhenius formula. Aside from Arrhenius fitting, the Elliott theory is also use to extract the exciton binding energy of halide perovskites in related literatures, where a large number of fitting parameters will get involved in the fitting process, usually leading to the deviation for the extracted *E*_b_ (*35*–*37*). Therefore, in this study, we only used the Arrhenius extracted *E*_b_ values to qualitatively compare the confinement effect between 2D tin halide perovskites with variable *n* values. In general, the decrease in exciton binding energy indicates a decreasing confinement effect with *n* values, which will lead to the suppression of the auger exciton recombination, an important loss mechanism and will limit exciton density (*38*).

**Fig. 2.**
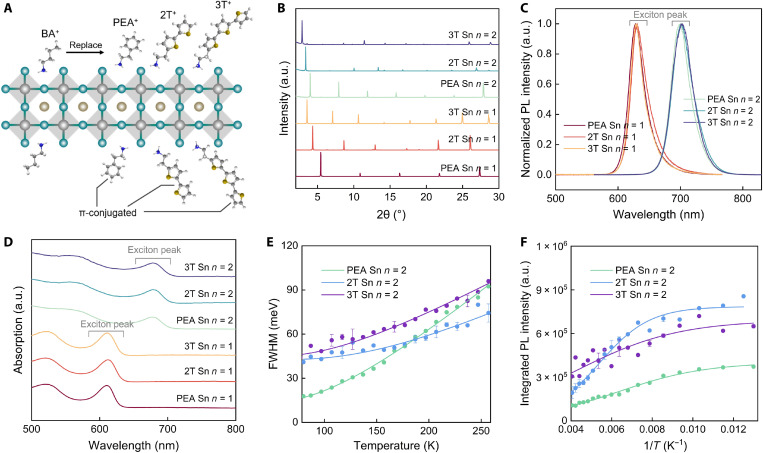
2D tin halide perovskites via ligand engineering. (**A**) Schematic diagram of ligand engineering in 2D tin halide perovskites (BA^+^, PEA^+^, 2T^+^, and 3T^+^). (**B**) PXRD patterns of L_2_MA_*n*−1_Sn*_n_*I_3*n*+1_ (L: PEA^+^, 2T^+^, and 3T^+^; *n*: 1 and 2). (**C**) PL and (**D**) UV-vis spectra of L_2_MA_*n*−1_Sn*_n_*I_3*n*+1_ (L: PEA^+^, 2T^+^, and 3T^+^; *n*: 1 and 2). (**E**) Exciton-phonon coupling and (**F**) exciton binding energies obtained from Arrhenius formula fitting of L_2_MASn_2_I_7_ (L: PEA^+^, 2T^+^, and 3T^+^).

From previous reports, the phase transition of 2D tin halide perovskite (*n* = 1) at low temperature [~257 K for (BA)_2_SnI_4_] can be inhibited via adopting PEA^+^ or hexylammonium ligand instead of BA^+^ (*31*). In addition, ligand engineering can also effectively tune the optical and electrical properties of RP halide perovskites (*14*, *39*). To improve the phase stability of 2D tin perovskite (*n* = 1) and gain more insights of the fundamental properties of RP tin halide perovskites, we selected three conjugated and bulky ligands (PEA^+^, 2T^+^, and 3T^+^) to synthesize corresponding 2D tin halide perovskites in this study (Fig. 2A). Different from BA^+^ and PEA^+^ ligands, the hydrophobicity of conjugated ligands 2T^+^ and 3T^+^ adds additional difficulty in synthesizing phase-pure RP halide perovskite bulk crystals simply in aqueous solvent. In view of this, we incorporated organic solvents with moderate polarity, e.g., ethanol or isopropanol, into HI/H_3_PO_2_ to balance the solubility of conjugated ligand cations and inorganic SnI_2_ salts, both of which would be easily dissolved in the mixed solvent upon heating (see Materials and Methods and table S1) (*40*). As a result, highly pure (2T)_2_MA_*n*−1_Sn*_n_*I_3*n*+1_ (*n* = 1 and 2, abbreviated as 2T Sn *n* = 1 and 2T Sn *n* = 2, respectively) and (3T)_2_MA_*n*−1_Sn*_n_*I_3*n*+1_ (*n* = 1 and 2, abbreviated as 3T Sn *n* = 1 and 3T Sn *n* = 2, respectively) crystals have been successfully synthesized. The corresponding PXRD patterns indicate that the interlayer distance gradually increases as the spacer ligand changes from PEA^+^ to 3T^+^. For example, for *n* = 1 perovskites, the interlayer distance is 1.62 nm for (PEA)_2_SnI_4_, 2.03 nm for (2T)_2_SnI_4_, and 2.48 nm for (3T)_2_SnI_4_; for *n* = 2 perovskite, the interlayer distance is 2.22 nm for (PEA)_2_MASn_2_I_7_, 2.64 nm for (2T)_2_MASn_2_I_7_, and 3.06 nm for (3T)_2_MASn_2_I_7_ (fig. S8 and tables S7 to S12).

These 2D tin halide perovskites containing conjugated ligands exhibit similar optical properties to the BA^+^ analogs, as revealed by the PL and UV-vis absorption spectra (Fig. 2, C and D). As expected, the phase transition occurring in (BA)_2_SnI_4_ at 257 K is inhibited in (PEA)_2_SnI_4_, (2T)_2_SnI_4_, and (3T)_2_SnI_4_ perovskites, evidenced by the vanishing of the sharp blue shift in temperature-dependent PL spectra (fig. S9). In addition, via extracting the FWHM and integral area from the temperature-dependent PL spectra, the exciton-phonon coupling strength and effective exciton binding energy of L_2_MA_*n*−1_Sn*_n_*I_3*n*+1_ (L = PEA^+^, 2T^+^, and 3T^+^; *n* = 1 and 2) were calculated. With the increase in the ligand size (from PEA^+^ to 3T^+^), the exciton-phonon coupling strength for *n* = 2 tin halide perovskites decreases from 198 to 142 meV, lower than *n* = 1 tin halide perovskites [405 meV for (PEA)_2_SnI_4_, 434 meV for (2T)_2_SnI_4_, and 354 meV for (3T)_2_SnI_4_; Fig. 2E, figs. S10 and S11, and table S6], which is consistent with the *n*-dependent results for BA tin halide perovskites (Fig. 1H). Estimated Arrhenius extracted exciton binding energies of these three types of perovskites were determined to be 40 meV for (PEA)_2_MASn_2_I_7_, 69 meV for (2T)_2_MASn_2_I_7_ and 34 meV for (3T)_2_MASn_2_I_7_, which are also lower than those of *n* = 1 tin halide perovskites [49 meV for (PEA)_2_SnI_4_, 158 meV for (2T)_2_SnI_4_, and 52 meV for (3T)_2_SnI_4_] (Fig. 2F, fig. S12, and table S6). The above comparison indicates that high–*n* value tin halide perovskites have smaller exciton-phonon coupling strength and exciton binding energies (Fig. 2F and fig. S12).

**Fig. 3.**
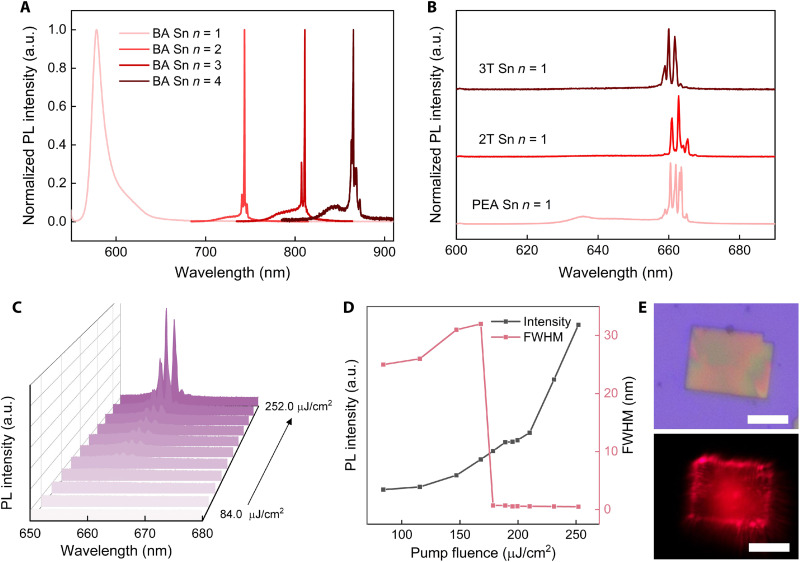
Lasing behaviors of 2D tin halide perovskites at 83 K via *n* value and ligand engineering. (**A**) Lasing PL spectra of (BA)_2_MA_*n*−1_Sn*_n_*I_3*n*+1_ (*n* = 1 to 4): (BA)_2_SnI_4_ at 415 μJ/cm^2^, (BA)_2_MASn_2_I_7_ at 189 μJ/cm^2^, (BA)_2_MA_2_Sn_3_I_10_ at 164 μJ/cm^2^, and (BA)_2_MA_3_Sn_4_I_13_ at 158 μJ/cm^2^. (**B**) Lasing spectra for (PEA)_2_SnI_4_ at 129 μJ/cm^2^, (2T)_2_SnI_4_ at 210 μJ/cm^2^, and (3T)_2_SnI_4_ at 252 μJ/cm^2^. (**C**) Lasing PL spectra of (3T)_2_SnI_4_ with pump fluence increasing from 84.0 to 252.0 μJ/cm^2^. (**D**) Pump fluence dependent PL intensity and FWHM of (3T)_2_SnI_4_. (**E**) Optical and lasing images of a (3T)_2_SnI_4_ thin flake. Scale bars, 5 μm.

Benefited from the direct bandgap property, low toxicity, and highly tunable emission wavelength, mechanically exfoliated RP tin halide perovskite thin flakes are ideally suited for developing efficient cavity-free microlasers. With the sample temperature cooling down to 83 K, optically pumped lasing was achieved in (BA)_2_MA_*n*−1_Sn*_n_*I_3*n*+1_ (*n* = 2 to 4) exfoliated flakes (Fig. 3A and fig. S13), where the edges of the exfoliated crystals act as the resonance cavity and the crystals themselves as the active gain material. The thickness of the crystals for lasing is determined as 335 to 368 nm (for *n* = 4; fig. S14) larger than the wavelength λ of the lasing light within the crystal (λ = 850 nm/*n*_refractive_, where *n*_refractive_ is the refractive index of the crystal; *n*_refractive_ ~ 3) (*41*), which ensures the efficient waveguide during the pumping process. This is the first report that lasing has been achieved in exfoliated 2D tin halide perovskite flakes without external cavity, while Ding *et al.* (*40*) and Alvarado-Leaños *et al.* (*42*) only achieved amplified spontaneous emission (ASE) in 2D tin halide perovskites when external resonant cavities were absent. Because of the self-absorption effect, the lasing peak of (BA)_2_MA_*n*−1_Sn*_n_*I_3*n*+1_ (*n* = 2 to 4) is red-shifted compared with their spontaneous emission spectra (fig. S15) (*19*). The absence of lasing in (BA)_2_SnI_4_ is probably caused by the phase transition at 257 K, which makes it difficult to conclude whether *n* = 1 tin halide perovskites can generate lasing or not. As verified above (fig. S9), there was no phase transition for 2D L_2_SnI_4_ (L = PEA^+^, 2T^+^, and 3T^+^) during the cooling process (from room temperature to 77 K). As a result, the optically pumped lasing was achieved from all these three 2D tin halide perovskites (at 83 K; Fig. 3B). In comparison, for RP lead perovskite thin flakes, the lasing signal can only be observed from samples with *n* > 1, and the lasing could not be optically pumped in *n* = 1 lead halide perovskites such as (2T)_2_PbI_4_ and (3T)_2_PbI_4_, even under a large pump fluence of 813 μJ/cm^2^ (83 K; fig. S16). The difference in lasing capability between tin perovskite (*n* = 1) and lead perovskite (*n* = 1) highlights the likelihood that tin halide RP perovskites are better material candidates than their lead counterparts for high-performance microlasers. The fluence-dependent PL emission of (3T)_2_Sn_4_ in Fig. 3, C and D resembles those of BA tin halide perovskites (fig. S13). According to our measurements, the threshold of (PEA)_2_SnI_4_ thin flake is ~117.2 μJ/cm^2^, and the quality factor *Q* (*Q* = λ/δλ, where λ refers to the lasing peak wavelength and δλ is the FWHW of the lasing peak) is 1835 (fig. S17). For (2T)_2_SnI_4_, the threshold is ~147.0 μJ/cm^2^, and the quality factor *Q* is 1506 (fig. S18). For (3T)_2_SnI_4_, the threshold is ~178.5 μJ/cm^2^, and the quality factor *Q* is 1294 (Fig. 3, C and D, and fig. S19). The difference in defect density makes it challenging to establish the correlation between the lasing threshold and the organic ligands in *n* = 1 tin halide perovskites (fig. S20) (*31*, *33*). Multiple sharp lasing peaks were observed for all these microlasers. From the optical image and finite-difference time-domain simulations under stimulation, the lasing should be caused by the resonant mode of an edge-confined in-plane optical microcavity (Fig. 3E and fig. S21).

**Fig. 4.**
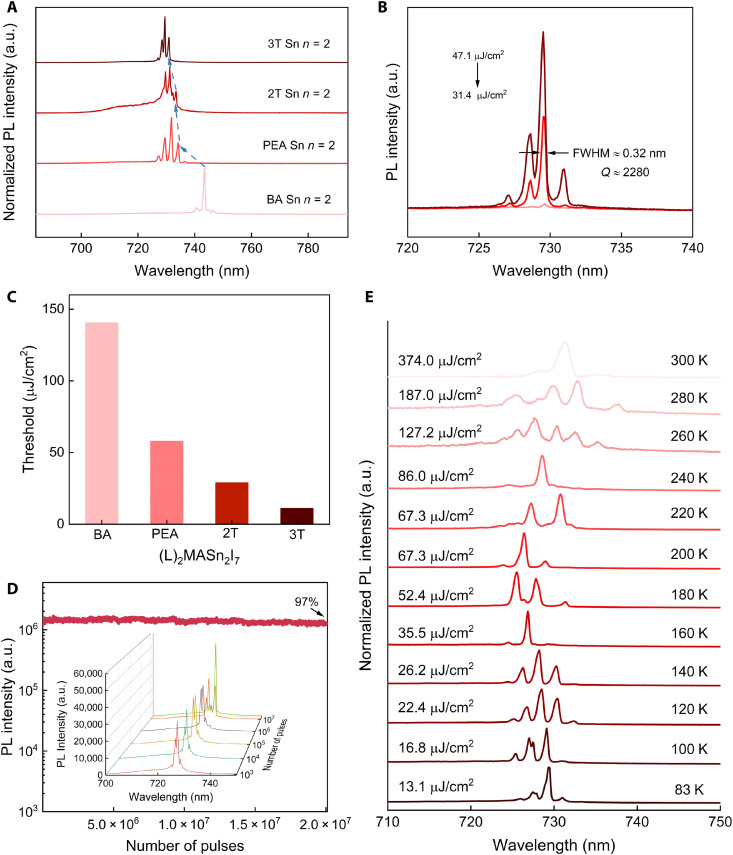
Lasing stability of 2D tin halide perovskites (*n* = 2). (**A**) Lasing PL spectra of (L)_2_MASn_2_I_7_ (L^+^: BA^+^, PEA^+^, 2T^+^, and 3T^+^) at 83 K. (**B**) Lasing spectra of (3T)_2_MASn_2_I_7_ with pump fluence increasing from 31.4 to 47.1 μJ/cm^2^ at 83 K. (**C**) Lasing threshold comparison of different 2D tin halide perovskites (*n* = 2) at 83 K (these threshold values are adopted from the lowest values in figs. S13 and S23 to S25). (**D**) The stability and involution of lasing spectra (inset) of the (3T)_2_MASn_2_I_7_ thin flake at 83 K with 200 s of acquisition time at the repetition rate of 100 kHz. (**E**) Temperature-dependent lasing PL spectra from 83 K to 300 K of (3T)_2_MASn_2_I_7_ thin flake.

The effect from ligand engineering on the lasing performance is further elaborated by comparing different 2D tin halide perovskites (L)_2_MASn_2_I_7_ (L = BA^+^, PEA^+^, 2T^+^, or 3T^+^). Because of the significant impact of the organic ligand on the inorganic framework, the lattice constants of 2D tin halide perovskites decrease by different degrees at low temperature, and the reduction of lattice constant enhances the interaction between the 5s orbital of Sn and the 5p orbital of I, so the perovskites with different ligands have different degrees of redshift at low temperature (fig. S22) (*23*). In this regard, the fine-tuning of the lasing spectra of 2D tin halide perovskites becomes possible, whose highest lasing peak shifts from 743.4 nm [(BA)_2_MASn_2_I_7_] to 728.5 nm [(3T)_2_MASn_2_I_7_] (Fig. 4A). Moreover, the lasing from 2D tin halide perovskites with conjugated ligands has a relatively high *Q*, which reaches 3046 for (2T)_2_MASn_2_I_7_ and 2280 for (3T)_2_MASn_2_I_7_ (Fig. 4B and figs. S23 to S25). In addition, the lasing emission from (3T)_2_MASn_2_I_7_ is highly polarized, further confirming the lasing behaviors rather than ASE (fig. S26). In addition to high *Q*, low lasing threshold is also highly desired to develop high-performance lasers. For these 2D tin perovskite flakes, since the shape and thickness of exfoliated flakes are random, the self-formed optical resonators will be different, leading to different degrees of energy loss, so there is a fluctuation in the lasing threshold. By comparing the lasing thresholds of 2D tin halide perovskites (*n* = 2), it is found that the lasing threshold is intimately related to the spacer ligands. The similar PL shape of L_2_MASn_2_I_7_ at 77 K, without any observable lower-energy defect peaks, enables a direct correlation between the lasing threshold and organic ligands (fig. S22). Among these compounds, 2D tin halide perovskite (*n* = 2) with larger ligands [(3T)_2_MASn_2_I_7_] has the lowest lasing threshold, which is 11.2 μJ/cm^2^ (at 83 K), benefiting from the lower exciton-phonon coupling strength and improved stability (including structural, photo, and environmental stability) (Fig. 4C).

As mentioned above, we obtained lasing signals in numerous mechanically exfoliated 2D tin-based perovskite samples, which could only be observed from samples with *n* ≥ 2 in lead-based ones. Besides, these cavity-free microlasers exhibited high lasing stability, e.g., both of (BA)_2_MASn_2_I_7_ and (3T)_2_MASn_2_I_7_ show no degradation in lasing intensity after 8 × 10^6^ pulses (Fig. 4D and figs. S27 and S28). In addition, the lasing emission of (3T)_2_MASn_2_I_7_ can be maintained from 83 to 300 K with increasing lasing threshold (Fig. 4E and figs. S25, S29, and S30) due to the enhancement of the exciton-phonon coupling and the accompanying energy loss. The realization of room-temperature lasers with high stability is a giant leap forward in 2D perovskite lasers, based on which continuous-wave lasers and electrically pumped lasers can be envisioned in the future.

## DISCUSSION

Our study demonstrates the controllable synthesis and fundamental study of a family of lead-free 2D semiconductors—RP tin halide perovskites with room-temperature and wide-range lasing capability. It is noted that further endeavors are still needed to optimize the cavity geometry (tailoring the size and shape of the thin flakes) while maintaining high phase purity and to gain deeper understanding of the correlations between crystal structure, lattice, carrier dynamics, and lasing performance. The ease of lasing from 2D tin halide perovskites and their high working stability highlight the prospect of RP tin perovskite flakes in future microlasers and integrated nanophotonics.

## MATERIALS AND METHODS

### Synthesis of 2D tin halide perovskites

Single crystals of 2D tin halide perovskites were synthesized by slow cooling method. After the growth solution was prepared following the recipe shown in table S1, the sealed glass vials containing corresponding precursors and solvents were placed in a muffle furnace and heated until all the solid precursors were completely dissolved. Thereafter, the solution was cooled to room temperature at a constant cooling rate of 2°C/hour. The as-synthesized crystals were collected, dried, and stored in a nitrogen glove box for subsequent further use. To minimize the production of Sn^4+^, weighing of the solid precursors, collection, and drying of the crystals were all performed in the nitrogen glovebox.

### PXRD and single-crystal measurements

The PXRD measurement was performed by Bruker D8 Advance instrument, scanning at 0.02° per step using a copper target. All the single crystals were completely analyzed using a Bruker AXS D8 Venture diffractometer with a high-intensity diamond-Cu/Mo hybrid dual-microfocal x-ray tube (Mo Kα radiation, λ = 0.71073 Å), triumph curved graphite crystal for monochromatization, and a Photon III charge-integrating pixel array detector with 10 × 14 cm^2^. Oxford Cryosystems low-temperature devices are included in the advanced single-crystal XRD equipment. All data collecting, reflection indexing and processing, files scaling, and correction for absorption were completed using APEX4 software. The space groups were assigned, and structures were resolved directly using XPREP as part of the SHELXTL and OLEX2 software suite. The method of full matrix least squares against *F*^2^ with all reflections was used for further refinement through Shelxl2018 with the graphical interface SHELXTL. These structures were verified by virtue of the ADDSYM algorithm from PLATON software. H atoms connected to carbon and nitrogen atoms were positioned geometrically and forced to ride on their parent atoms unless otherwise stated. The tables S2 to S5 and S7 to S12 provide a summary of detailed crystal parameters.

### Optical images, PL images, and spectra measurements

The bright-field optical images were collected by a Nikon LV100ND microscope. For PL images, samples were excited with a mercury fluorescence light source (C-LHGFI HG LAMP). The fluorescence filter cube contains a bandpass filter (330 to 385 nm) for excitation, a dichroic mirror (cutoff wavelength, 400 nm) for light splitting, and a filter (long pass, 410 nm) for emission. The steady-state PL spectra were collected by a Princeton Instruments spectrometer (HRS-300S).

### UV-vis absorption spectra measurements

The UV-vis absorption (abs.) spectra were collected by CRAIC 20/30PV Pro instrument. After collecting the transmission (*T*) and reflection (*R*) spectra at the same location, abs. = −log[*T*/(1 − *R*)] was used to transform the relationship between the absorption spectrum and wavelength.

### AFM and LFM

The AFM and LFM measurement was conducted under N_2_ atmosphere by using Cypher ES instrument at room temperature. The AFM is under AC Air Topography mode, and the LFM is under lateral mode.

### Temperature-dependent PL measurements

The temperature-dependent PL measurement was conducted by combining a Linkam THMS600 stage with the Witec system. Before starting a cooling experiment, we need to purge the air from the stage chamber with dry nitrogen to remove the water and oxygen in the air. The cooling rate is set 20°C/min. We used the 50× long-distance objective lens for signal collecting. The exciton-phonon coupling strength is extracted from the temperature-dependent PL spectra. When the defect emission becomes significant and the experimental PL spectra of 2D tin halide perovskites have two clear peaks, the excitonic PL emission and the defect emission would be split using Gaussian fitting, and the FWHW of the symmetric excitonic PL peak would be used to extract the exciton-phonon coupling strength.

### Lasing measurement

We used a home-built microscope setup for lasing PL spectroscopy measurement. The fundamental 1030 nm beam from Yb: KGW laser (Carbide, Light Conversion Ltd.; 100 kHz) with a temporal resolution of 150 fs was focused onto a barium metaborate (BBO) crystal to generate a high-power 515 nm excitation light by second harmonic generation. The pump beam was focused onto mechanically exfoliated samples by microscope with a 50× objective. In addition, an optical lens with a focal length of 40 cm was placed on the light path to increase the light spot size to ~100 μm^2^. The collection and analysis of PL were conducted by liquid nitrogen cooled detectors (PyLon 100B, Princeton Instruments) or semiconductor refrigeration detectors (PIXIS, Princeton Instruments). The PL spectra collection time is 10 ms, and there are 1000 pulses in a row for each measurement. The lasing measurement was performed in vacuum.

### Time-resolved photoluminescence

The TRPL of samples was recorded by the time-correlated single photon counting module (SPC-130NX) and a photomultiplier (HPM-100-07). To measure the TRPL from 2D tin and lead halide perovskites, samples were excited by a 515 nm femtosecond laser with the repetition frequency of 500 KHz. This measurement was performed in vacuum.
