# Supplementary material for: Phase-pure 2D tin halide perovskite thin flakes for stable lasing
Source: Sci Adv. 2023 Aug 9;9(32):eadh0517. doi: 10.1126/sciadv.adh0517 (PMC11801371; doi:10.1126/sciadv.adh0517)
Supplement: Supplementary file 1 — Figs S1 to S30 Tables S1 to S10 Legends for data S1 to S8 [file sciadv.adh0517_sm.pdf]

Science Advances
AAAS

Supplementary Materials for

Phase-pure 2D tin halide perovskite thin flakes for stable lasing

Yahui Li *et al.*

Corresponding author: Haiming Zhu, hmzhu@zju.edu.cn; Enzheng Shi, shienzheng@westlake.edu.cn

Sci. Adv. **9**, eadh0517 (2023)
DOI: 10.1126/sciadv.adh0517

The PDF file includes:

Figs. S1 to S30
Tables S1 to S10
Legends for data S1 to S8

Other Supplementary Material for this manuscript includes the following:

Data S1 to S8

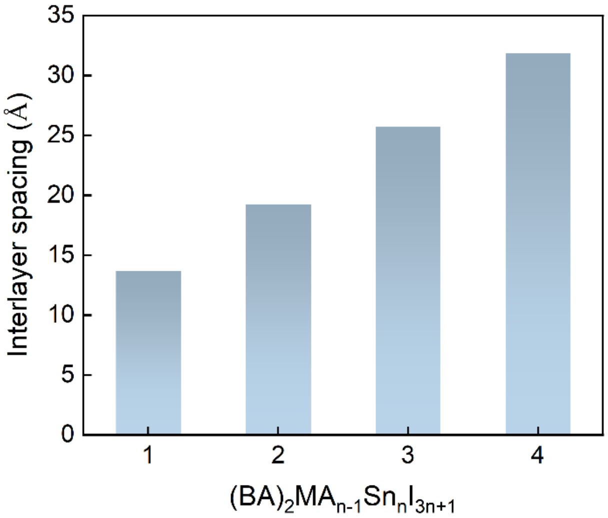

Fig. S1. The interlayer spacing of $(BA)_2MA_{n-1}Sn_nI_{3n+1}$ calculated from PXRD (n=1~4).

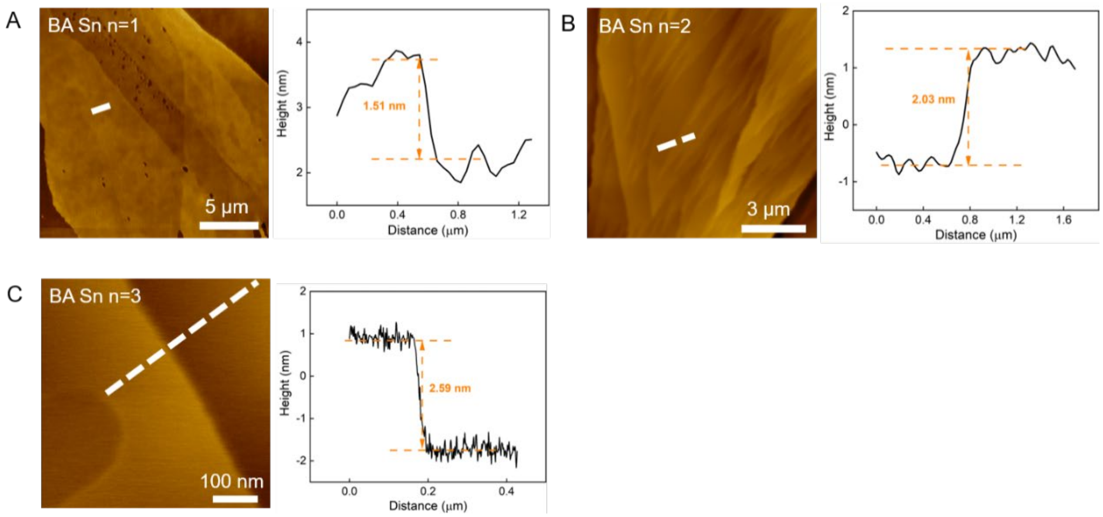

Fig. S2. AFM images and height profiles of $(BA)_2MA_{n-1}Sn_nI_{3n+1}$ (A: n=1, B: n=2, C: n=3).

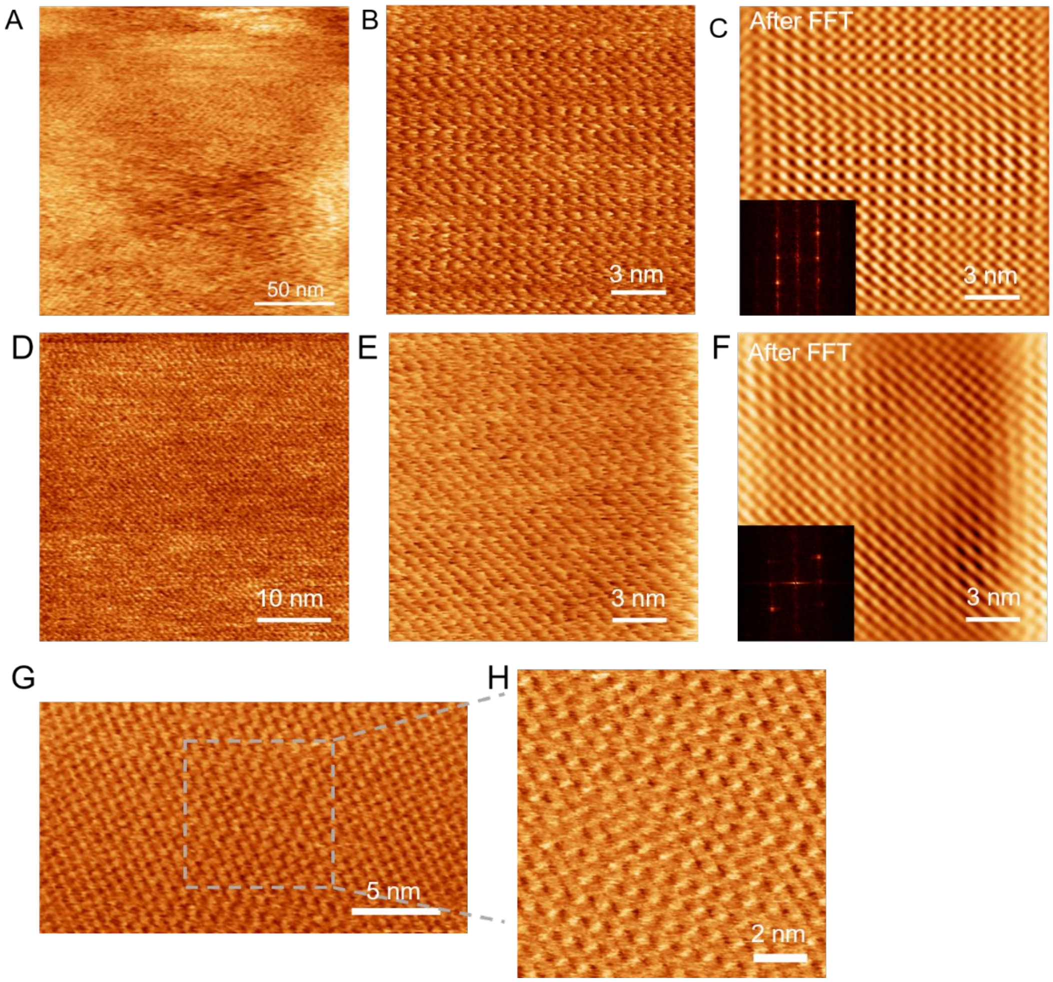

Fig. S3. High-resolution AFM images of $(BA)_2MA_{n-1}Sn_nI_{3n+1}$ (n=1~4) exfoliated flakes using LFM mode. (A) $(BA)_2SnI_4$. The absence of Fourier filtered images is due to the more unstable and softer structure. (B, C) High-resolution and Fourier filtered image of exfoliated $(BA)_2MASn_2I_7$. (D-F) High-resolution and Fourier filtered image of exfoliated $(BA)_2MA_2Sn_3I_{10}$. (G，H) High-resolution AFM image of exfoliated $(BA)_2MA_3Sn_4I_{13}$.

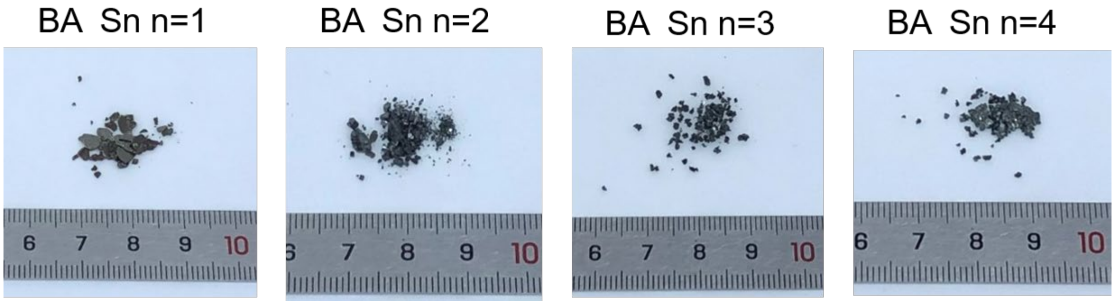

Fig. S4. Photo images of $(BA)_2MA_{n-1}Sn_nI_{3n+1}$ bulk crystals (n=1~4).

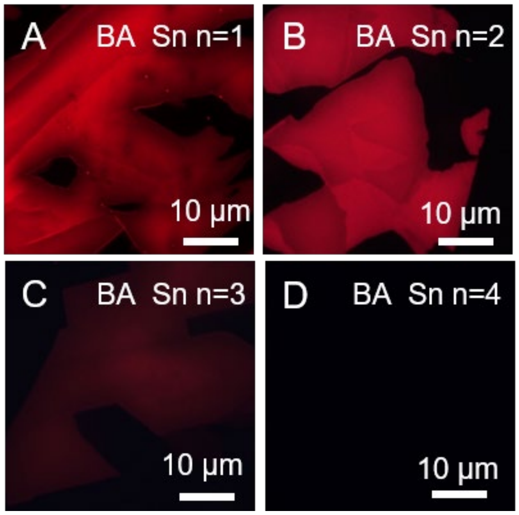

Fig. S5. PL images of mechanically exfoliated $(BA)_2MA_{n-1}Sn_nI_{3n+1}$ thin flakes (n=1~4). With the PL emission approaching to near-infrared range, the brightness of the PL emission of $(BA)_2MA_{n-1}Sn_nI_{3n+1}$ decreases.

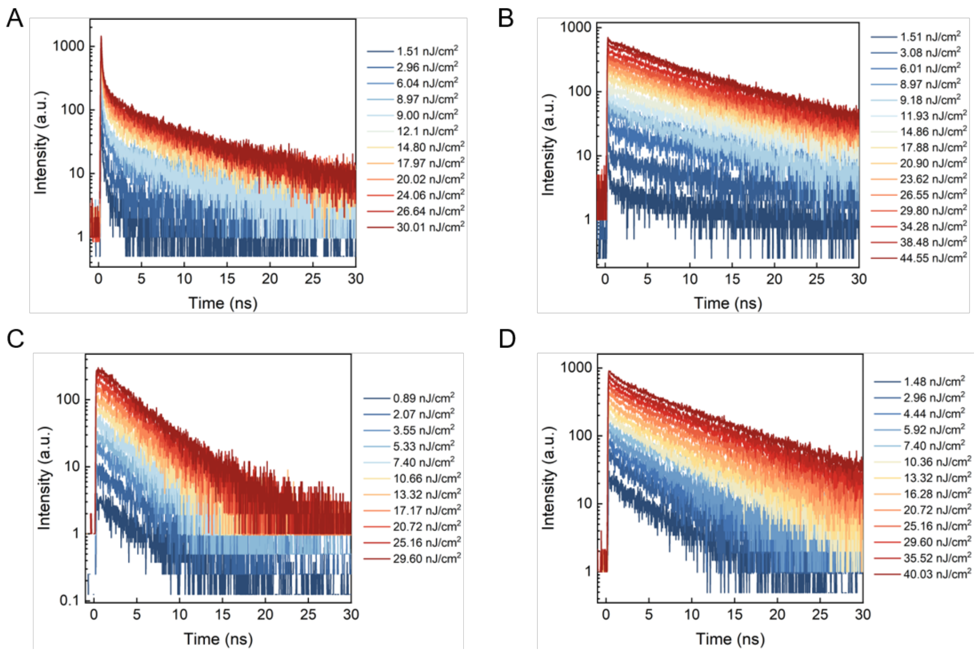

Fig. S6. PL decay traces at different laser power fluences of (A) $(BA)_2SnI_4$; (B) $(BA)_2MASn_2I_7$; (C) $(BA)_2MA_2Sn_3I_{10}$; (D) $(BA)_2MA_3Sn_4I_{13}$.

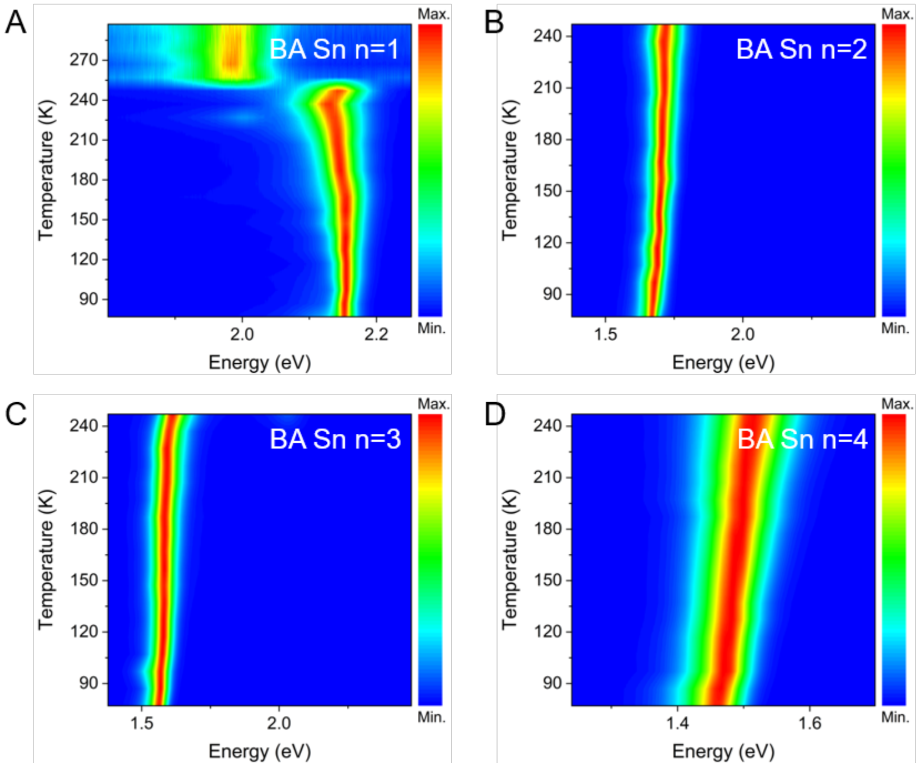

Fig. S7. Normalized temperature-dependent PL spectra of (A) $(BA)_2SnI_4$; (B) $(BA)_2MASn_2I_7$; (C) $(BA)_2MA_2Sn_3I_{10}$; (D) $(BA)_2MA_3Sn_4I_{13}$.

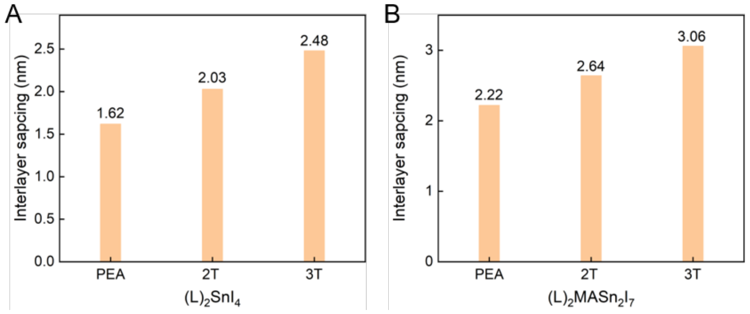

Fig. S8. The interlayer spacing of (A) $(L)_2SnI_4$ and (B) $(L)_2MASn_2I_7$ (L is PEA^+, $2T^+$, $3T^+$).

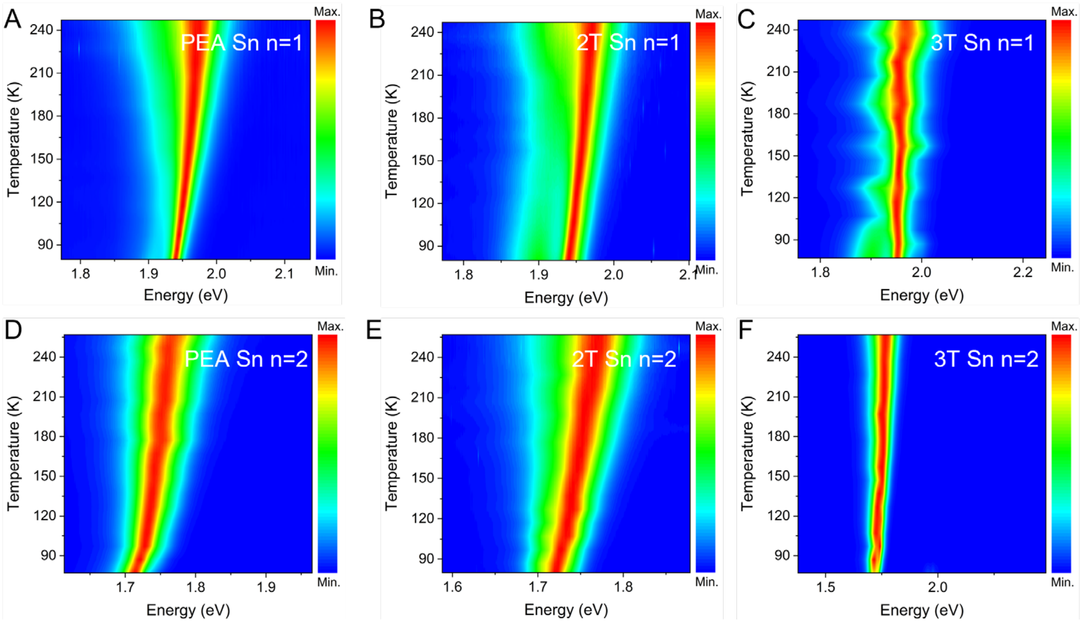

Fig. S9. Normalized temperature-dependent PL spectra of (A) $(PEA)_2SnI_4$, (B) $(2T)_2SnI_4$ and (C) $(3T)_2SnI_4$, (D) $(PEA)_2MASn_2I_7$, (E) $(2T)_2MASn_2I_7$ and (F) $(3T)_2MASn_2I_7$.

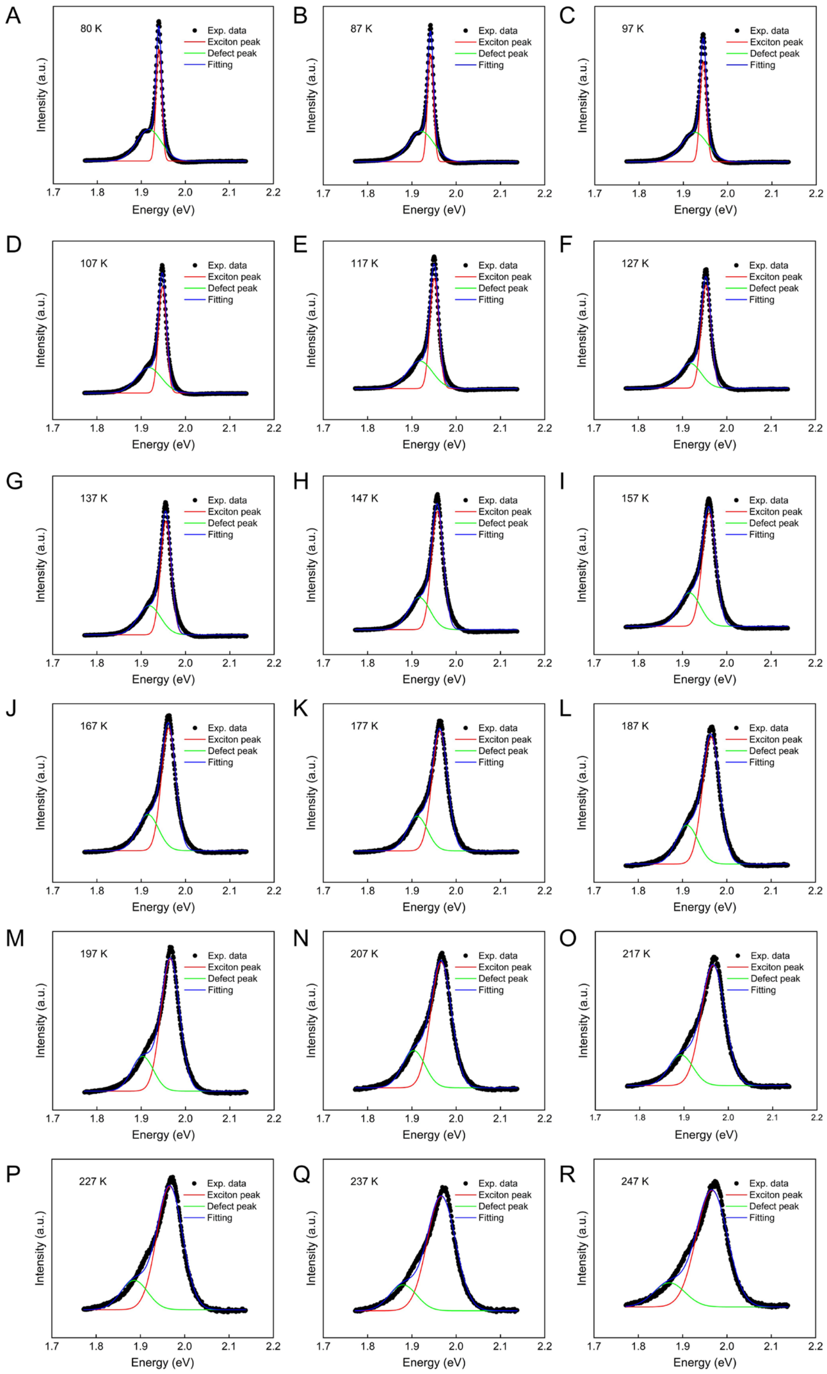

Fig. S10. Gaussian fitting of PL peaks of $(PEA)_2SnI_4$ at (A) 80 K, (B) 87 K, (C) 97 K, (D) 107 K, (E) 117 K, (F) 127 K, (G) 137 K, (H) 147 K, (I) 157 K, (J) 167 K, (K) 177 K, (L) 187 K, (M) 197 K, (N) 207 K, (O) 217 K, (P) 227 K, (Q) 237 K, (R) 247 K.

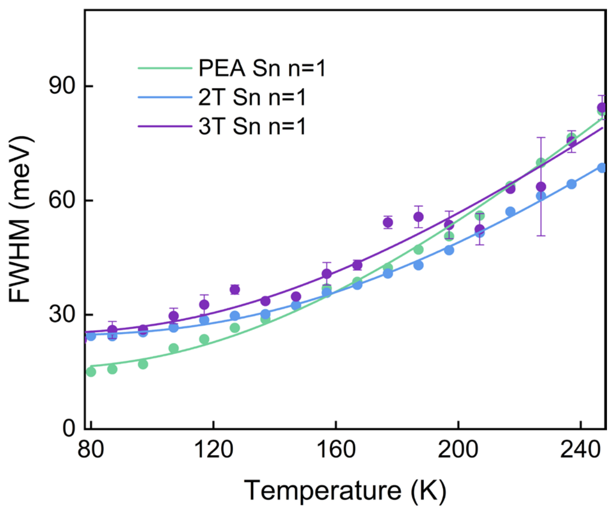

Fig. S11. Exciton-phonon coupling of $(L)_2SnI_4$ perovskites (L: PEA^+, $2T^+$, $3T^+$).

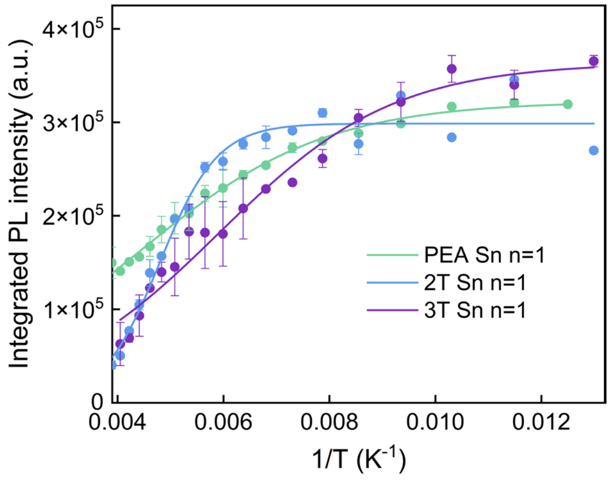

Fig. S12. Exciton binding energies of $(L)_2SnI_4$ (L: PEA^+, $2T^+$, $3T^+$).

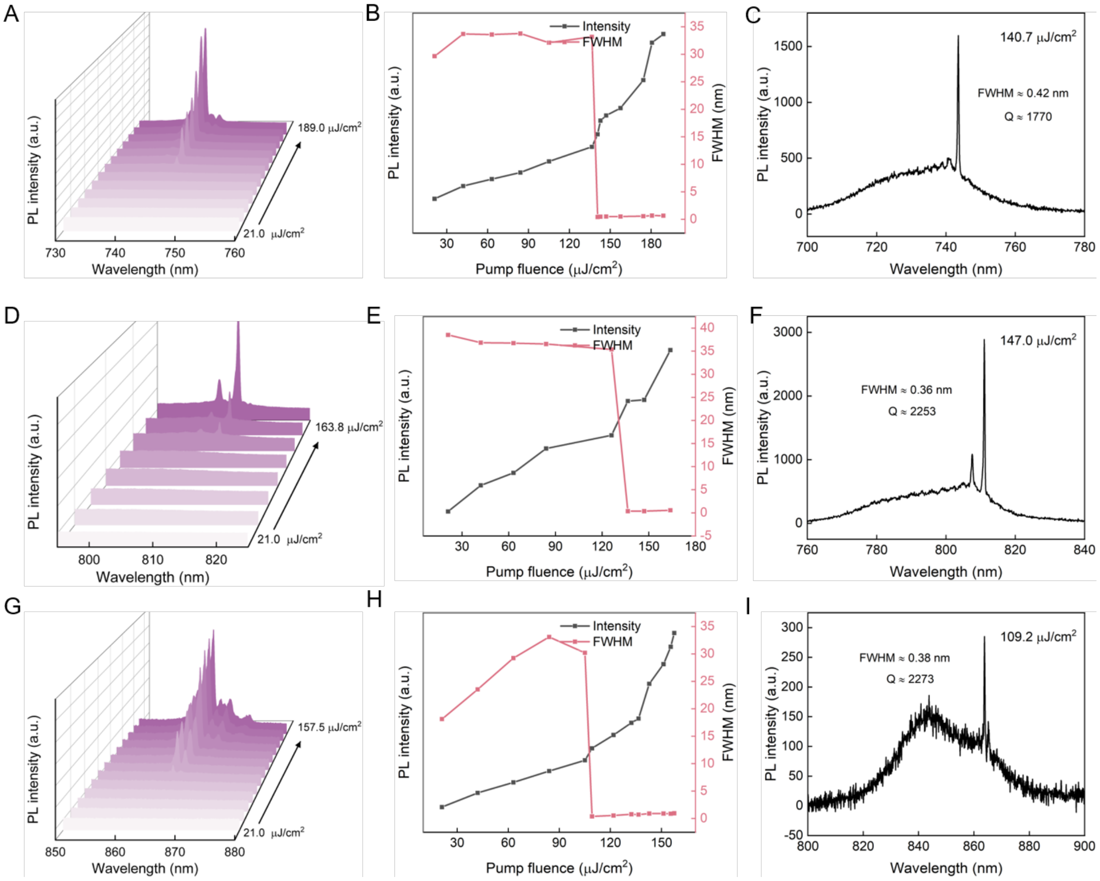

Fig. S13. Lasing characteristics of $(BA)_2MA_{n-1}Sn_nI_{3n+1}$ (n=2~4). Lasing characteristics of $(BA)_2MASn_2I_7$ (A-C): (A) lasing PL spectra with pump fluence increasing from 21.0 μJ/cm^2 to 189.0 μJ/cm^2, (B) pump fluence dependent PL intensity and FWHM at 83 K; (C) the FWHM of the lasing peak at 140.7 μJ/cm^2 is 0.42 nm, corresponding to a Q factor 1770. Lasing characteristics of $(BA)_2MA_2Sn_3I_{10}$ (D-F): (D) lasing PL spectra with pump fluence increasing from 21.0 μJ/cm^2 to 163.8 μJ/cm^2, (E) pump fluence dependent PL intensity and FWHM at 83 K; (F) the FWHM of the lasing peak at 147.0 μJ/cm^2 is 0.36 nm, corresponding to a Q factor of 2253. Lasing characteristics of $(BA)_2MA_3Sn_4I_{13}$ (G-I): (G) lasing PL spectra with pump fluence increasing from 21.0 μJ/cm^2 to 157.5 μJ/cm^2, (H) pump fluence dependent PL intensity and FWHM of $(BA)_2MA_3Sn_4I_{13}$; (I) the FWHM of the lasing peak at 109.2 μJ/cm^2 is 0.38 nm, corresponding to a Q factor of 2273.

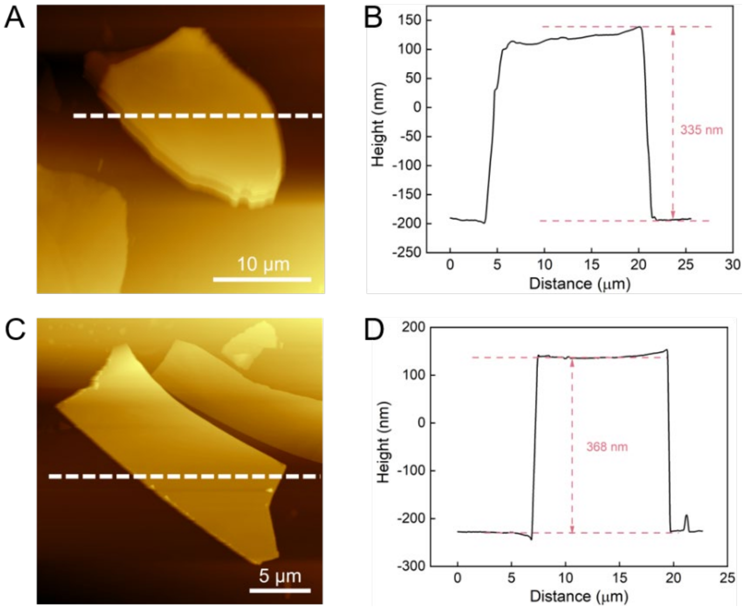

Fig. S14. AFM images and height profiles of two $(BA)_2MA_3Sn_4I_{13}$ thin flakes for lasing. (A, B) Flake-I. (C, D) Flake-II.

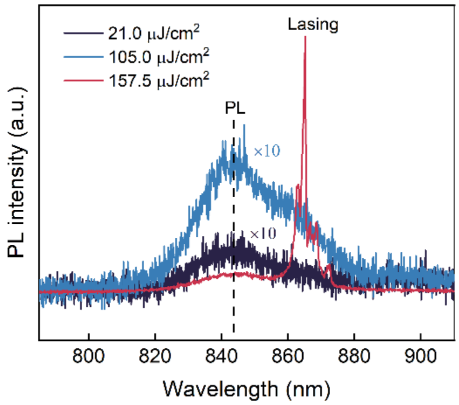

Fig. S15. Lasing PL spectra of $(BA)_2MA_3Sn_4I_{13}$ at a pump fluence of 21.0 μJ/cm^2, 105.0 μJ/cm^2, 157.5 μJ/cm^2, respectively.

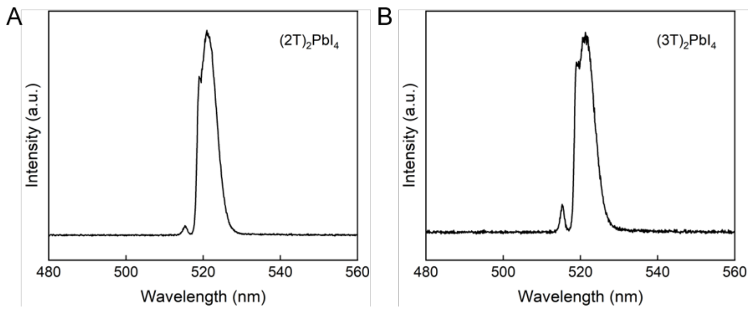

Fig. S16. PL spectra of 2D lead halide perovskites (n=1) at a pump fluence of 813 μJ/cm^2.
(A) $(2T)_2PbI_4$. (B) $(3T)_2PbI_4$

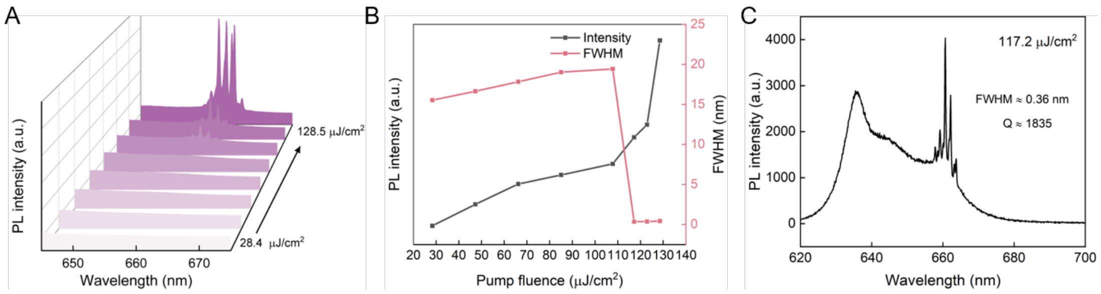

Fig. S17. Lasing characteristics of $(PEA)_2SnI_4$. PL spectra evolution with pump fluence increasing from 28.4 μJ/cm^2 to 128.5 μJ/cm^2 (A), pump fluence dependent PL intensity (B), and FWHM (C) at 83 K. The FWHM of the lasing peak at 117.2 μJ/cm^2 is 0.36 nm, corresponding to a Q factor of 1835.

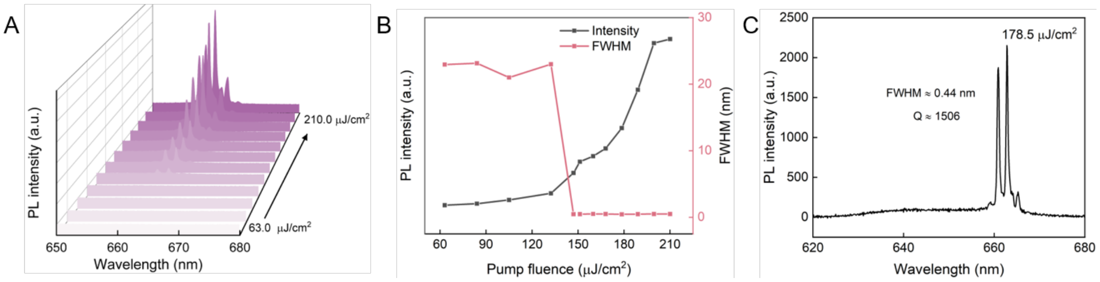

Fig. S18. Lasing characteristics of $(2T)_2SnI_4$. Lasing PL spectra with pump fluence increasing from 63.0 μJ/cm^2 to 210.0 μJ/cm^2 (A), pump fluence dependent PL intensity (B), and FWHM (C) at 83 K. The FWHM of the lasing peak at 178.5 μJ/cm^2 is 0.44 nm, corresponding to a Q factor of 1506.

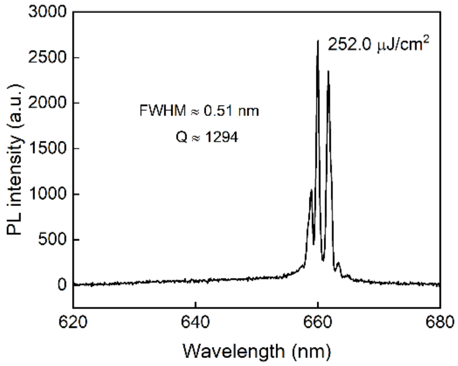

Fig. S19. PL lasing spectrum of a $(3T)_2SnI_4$ thin flake at 83 K under a pump fluence of 252.0 μJ/cm^2. The FWHM of the lasing peak is 0.51 nm, corresponding to a Q factor of 1294.

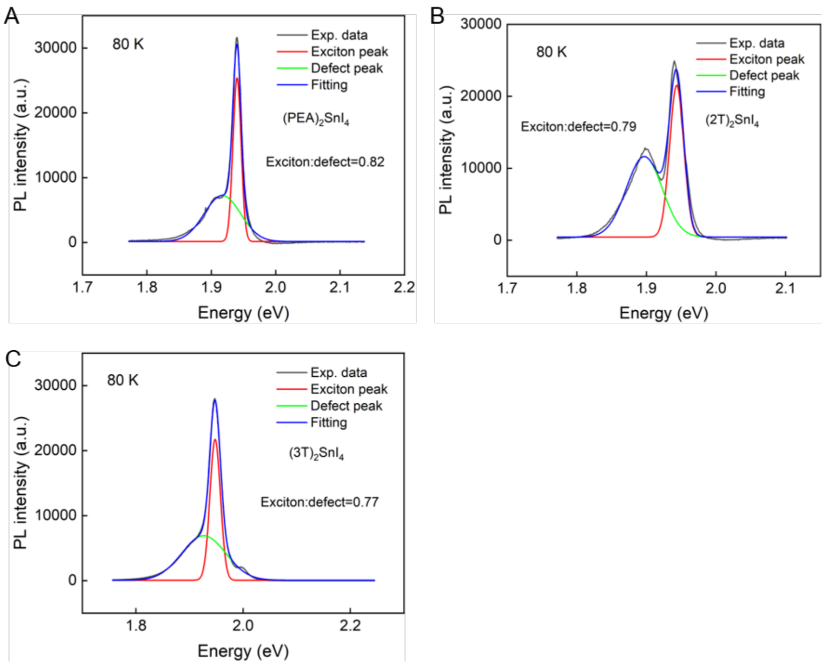

Fig. S20. Gaussian fitting of PL peaks of $(L)_2SnI_4$ (L: PEA^+, $2T^+$, $3T^+$). (A) Two Gaussian peaks fitting is applied to fit the PL peaks of $(PEA)_2SnI_4$ at 80 K. (B) Two Gaussian peaks fitting is applied to fit the PL peaks of $(2T)_2SnI_4$ at 80 K. (C) Two Gaussian peaks fitting is applied to fit the PL peaks of $(3T)_2SnI_4$ at 80 K.

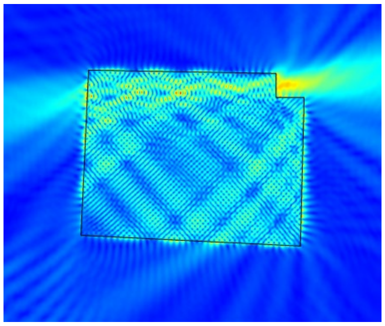

Fig. S21. Simulated electric field intensity distribution of $(3T)_2SnI_4$.

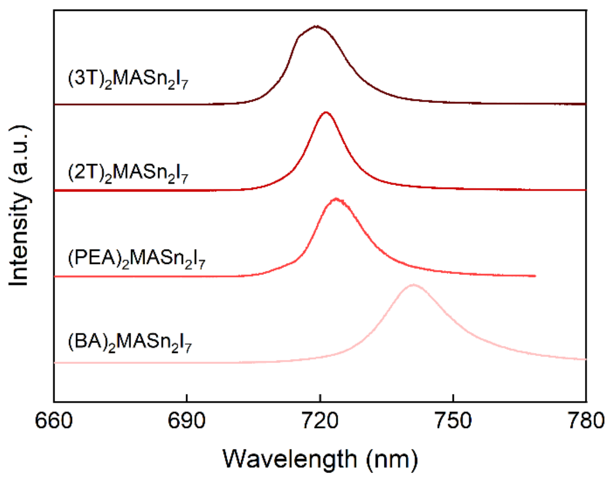

Fig. S22. PL spectra of $(L)_2MASn_2I_7$ at 77 K.

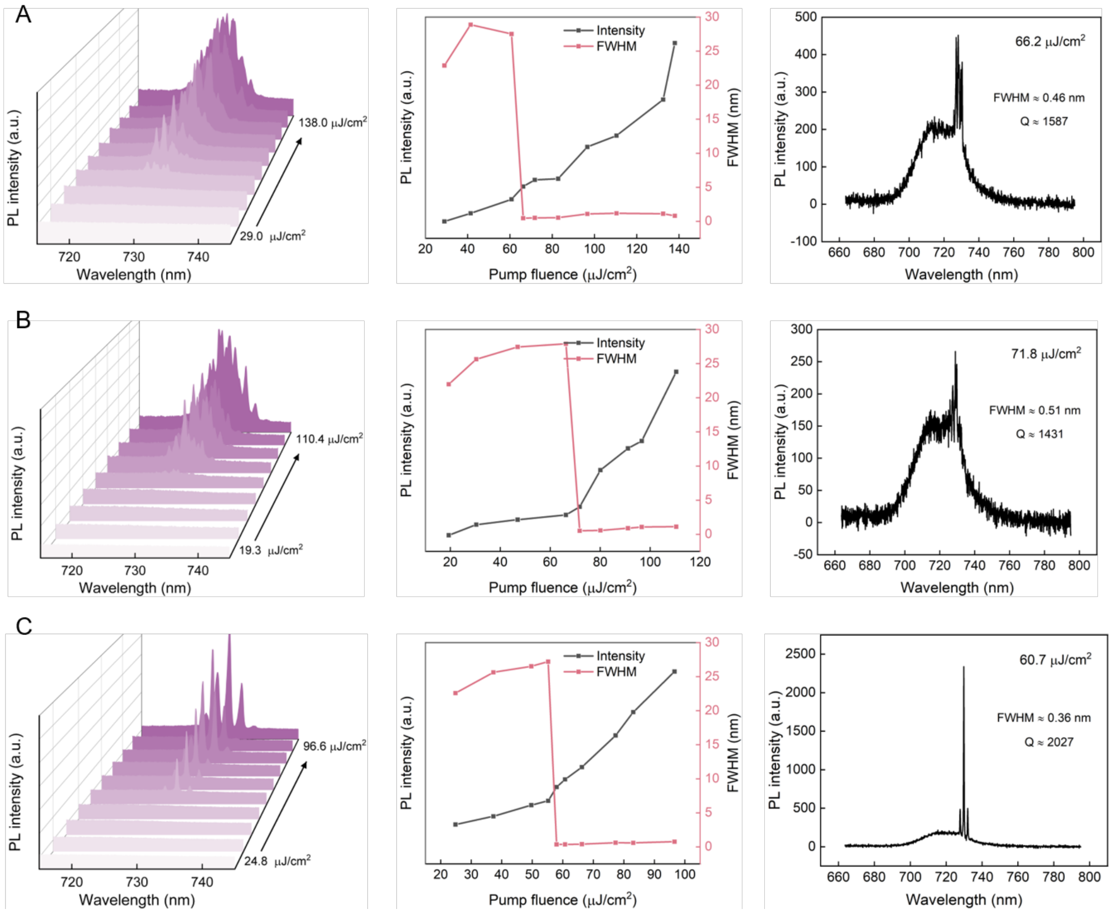

Fig. S23. Lasing tests from three different areas on the same $(PEA)_2MASn_2I_7$ thin flake. (A) PL spectra evolution with pump fluence increasing from 29.0 μJ/cm^2 to 138.0 μJ/cm^2, pump fluence dependent PL intensity, and FWHM at 83 K. The FWHM of the lasing peak at 66.2 μJ/cm^2 is 0.46 nm, corresponding to a Q factor of 1587. (B) PL spectra evolution with pump fluence increasing from 19.3 μJ/cm^2 to 110.4 μJ/cm^2, pump fluence dependent PL intensity, and FWHM at 83 K. The FWHM of the lasing peak at 71.8 μJ/cm^2 is 0.51 nm, corresponding to a Q factor of 1431. (C) PL spectra evolution with pump fluence increasing from 24.8 μJ/cm^2 to 96.6 μJ/cm^2, pump fluence dependent PL intensity, and FWHM at 83 K. The FWHM of the lasing peak at 60.7 μJ/cm^2 is 0.36 nm, corresponding to a Q factor of 2027.

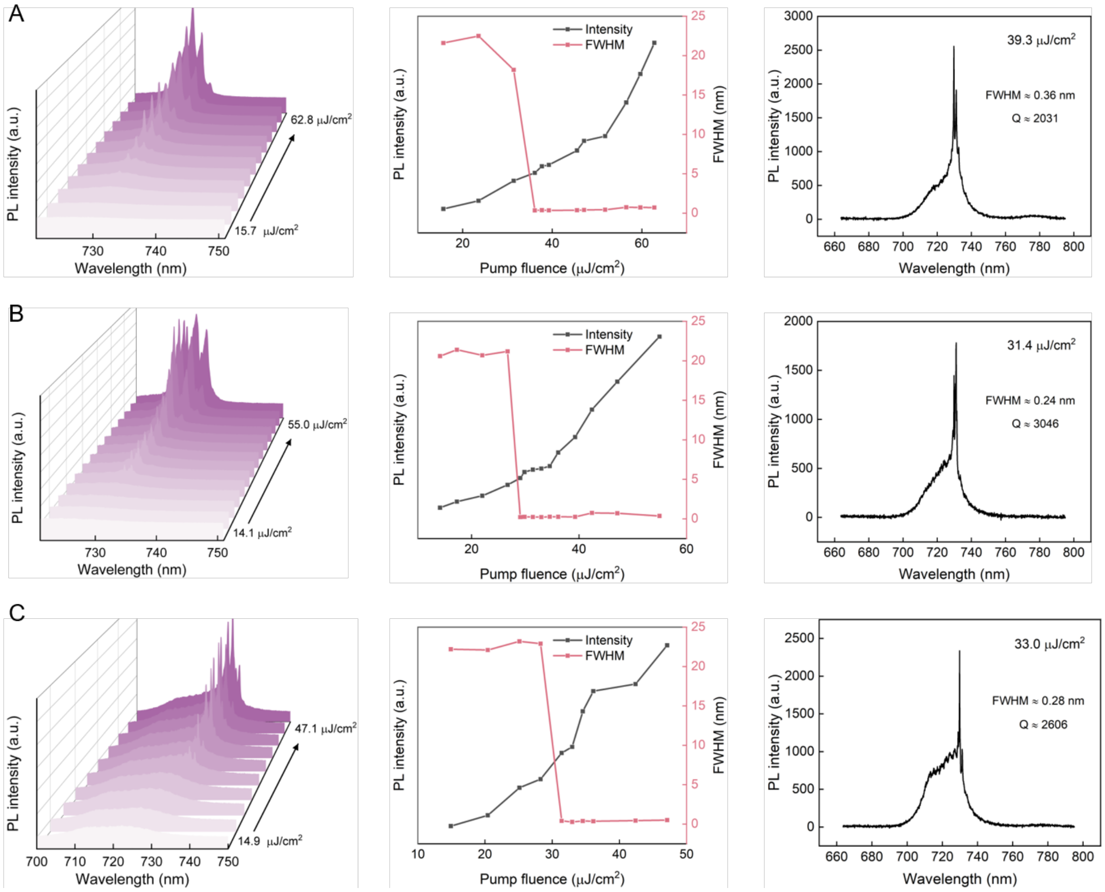

Fig. S24. Lasing tests from three different areas on the same $(2T)_2MASn_2I_7$ thin flake. (A) PL spectra evolution with pump fluence increasing from 15.7 μJ/cm^2 to 62.8 μJ/cm^2, pump fluence dependent PL intensity, and FWHM at 83 K. The FWHM of the lasing peak at 39.3 μJ/cm^2 is 0.36 nm, corresponding to a Q factor of 2031. (B) PL spectra evolution with pump fluence increasing from 14.1 μJ/cm^2 to 55.0 μJ/cm^2, pump fluence dependent PL intensity, and FWHM at 83 K. The FWHM of the lasing peak at 31.4 μJ/cm^2 is 0.24 nm, corresponding to a Q factor of 3046. (C) PL spectra evolution with pump fluence increasing from 14.9 μJ/cm^2 to 47.1 μJ/cm^2, pump fluence dependent PL intensity, and FWHM at 83 K. The FWHM of the lasing peak at 33.0 μJ/cm^2 is 0.28 nm, corresponding to a Q factor of 2606.

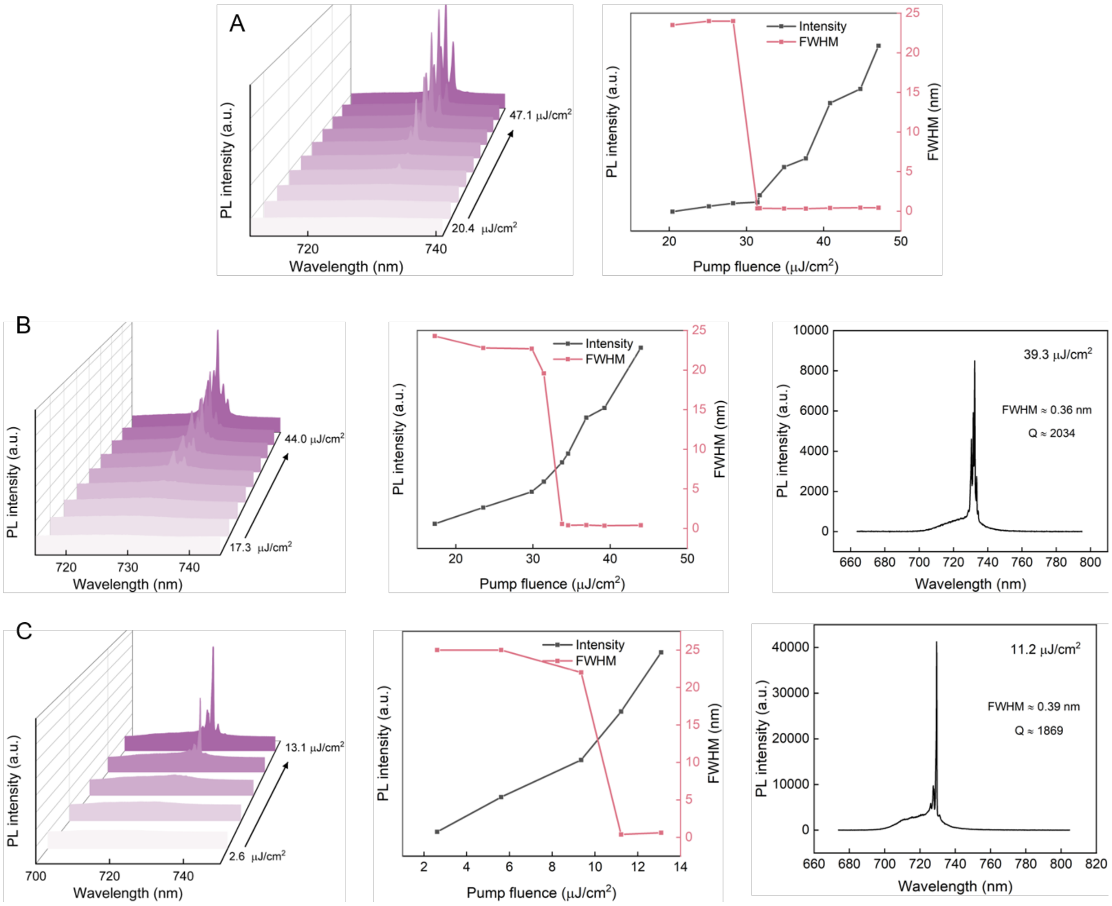

Fig. S25. Lasing tests from two different areas on the same $(3T)_2MASn_2I_7$ thin flake. (A) PL spectra evolution with pump fluence increasing from 20.4 μJ/cm^2 to 47.1 μJ/cm^2, pump fluence dependent PL intensity, and FWHM at 83 K. The lasing spectrum from this area is shown in Fig. 4B in the main text. (B) PL spectra evolution with pump fluence increasing from 17.3 μJ/cm^2 to 44.0 μJ/cm^2, pump fluence dependent PL intensity, and FWHM at 83 K. The FWHM of the lasing peak at 39.3 μJ/cm^2 is 0.36 nm, corresponding to a Q factor of 2034. Lasing tests from another $(3T)_2MASn_2I_7$ thin flake. (C) PL spectra evolution with pump fluence increasing from 2.6 μJ/cm^2 to 13.1 μJ/cm^2, pump fluence dependent PL intensity, and FWHM at 83 K. The FWHM of the lasing peak at 11.2 μJ/cm^2 is 0.39 nm, corresponding to a Q factor of 1869.

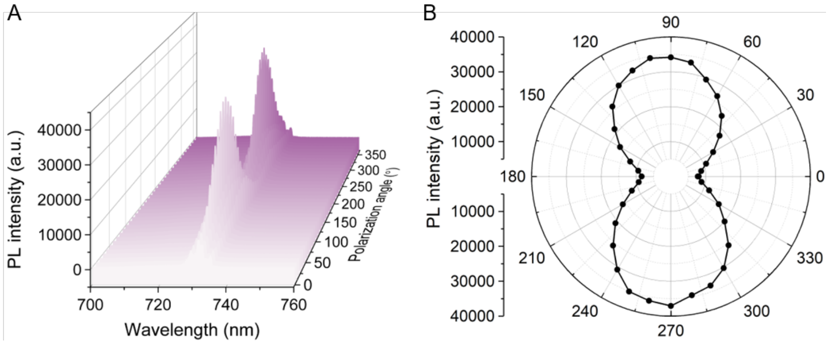

Fig. S26. Polarization characterization of lasing from $(3T)_2MASn_2I_7$. (A) Polarization-dependent PL spectra with the emission polarization oriented by each corresponding angle relative to the vertical direction. (B) Polar plot of the emission intensity.

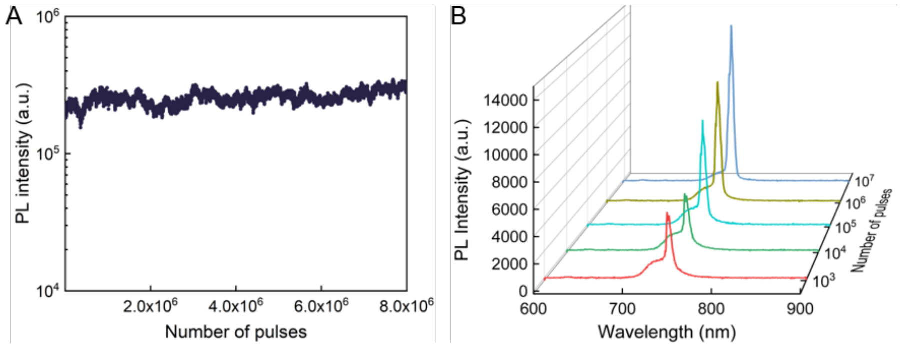

Fig. S27. Lasing stability (A) and lasing spectra (B) of $(BA)_2MASn_2I_7$ at 83 K.

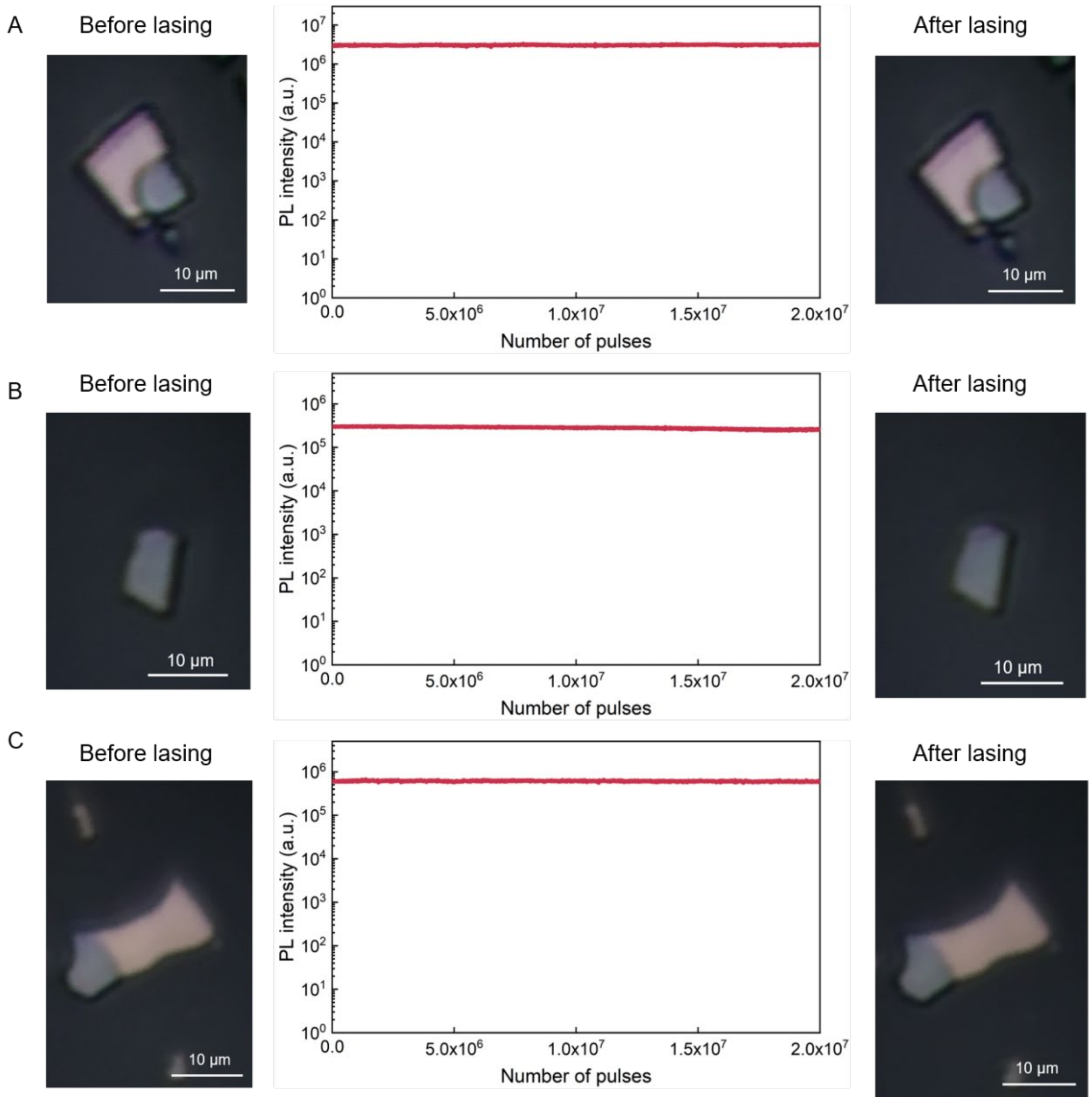

Fig. S28. Optical images of three individual $(3T)_2MASn_2I_7$flakes before and after lasing, as well as their lasing stability at 83 K. (A) Flake-I. (B) Flake-II. (C) Flake-III.

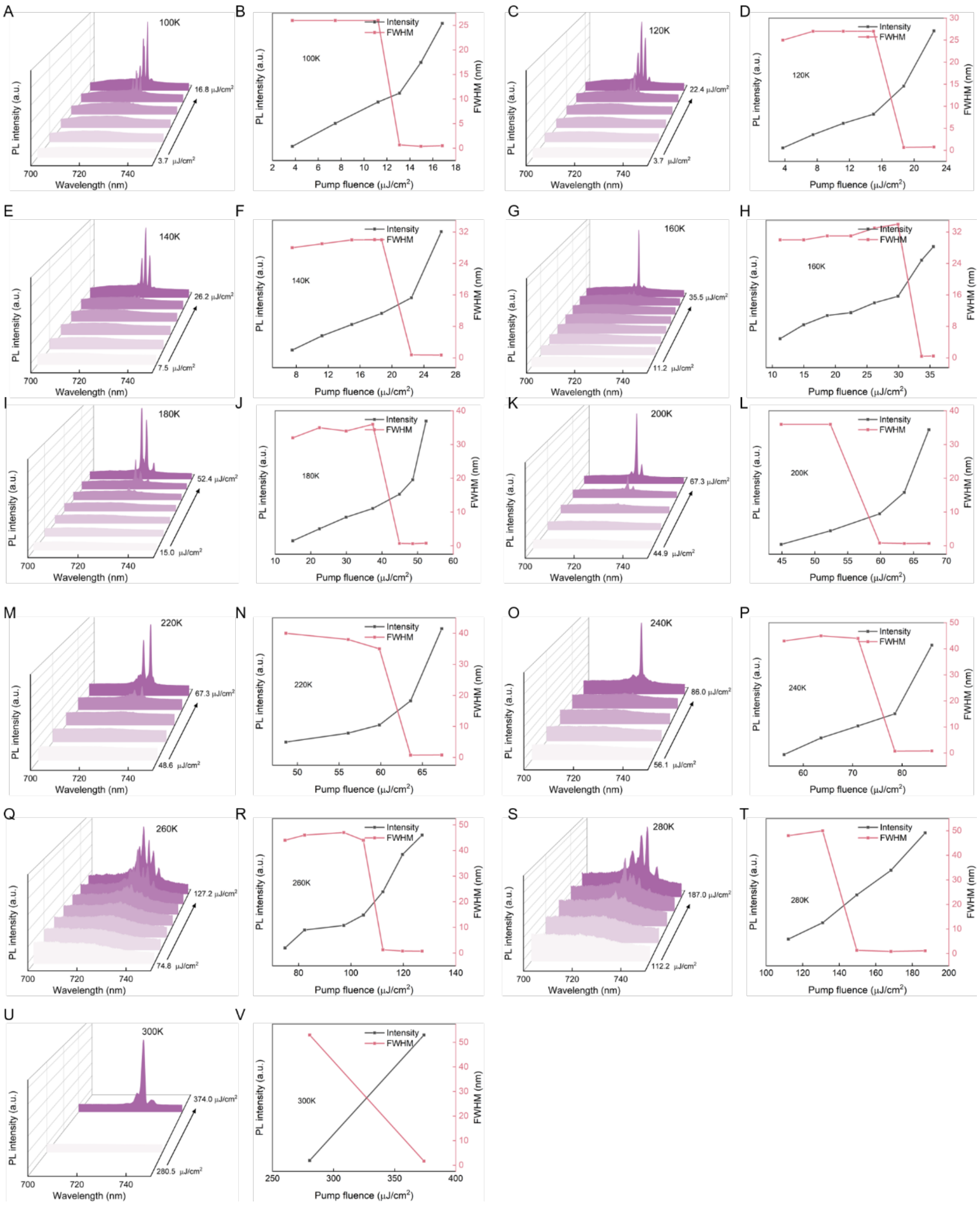

Fig. S29. The pump fluence dependence of PL spectra and FWHW of $(3T)_2MASn_2I_7$ at different measurement temperature. (A-B) 100 K. (C-D) 120 K. (E-F) 140 K. (G-H) 160 K. (I-J) 180 K. (K-L) 200 K. (M-N) 220 K. (O-P) 240 K. (Q-R) 260 K. (S-T) 280 K. (U-V) 300 K.

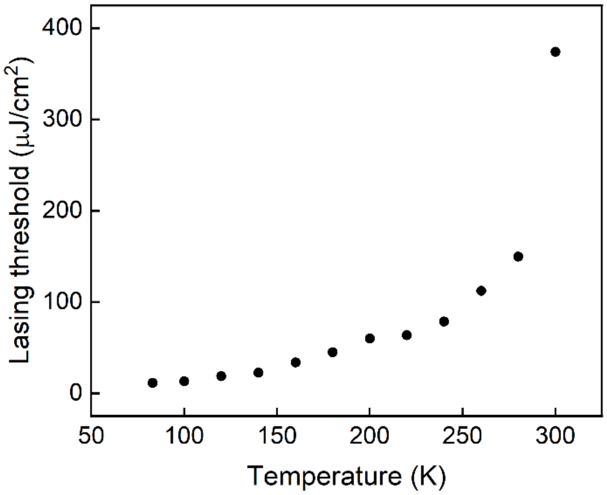

Fig. S30. Temperature dependence of lasing threshold of $(3T)_2MASn_2I_7$.

Table S1. Growth recipe for 2D tin halide perovskite bulk crystals

Single crystals	BAI (mmol)	PEAI (mmol)	2TI (mmol)	3TI (mmol)	MAI (mmol)	SnI_2 (mmol)	HI (mL)	H_3PO_2 (mL)	IPA (mL)	Ethanol (mL)
$(BA)_2SnI_4$	0.45					0.40	1	0.1		
$(BA)_2MASn_2I_7$	0.43				0.2	0.59	1	0.1		
$(BA)_2MA_2Sn_3I_{10}$	0.43				0.46	0.59	1	0.1		
$(BA)_2MA_3Sn_4I_{13}$	0.20				0.50	0.30	1	0.1		
$(PEA)_2SnI_4$		0.16				0.40	2	0.2		
$(PEA)_2MASn_2I_7$		0.17			0.4	0.40	2	0.2		
$(2T)_2SnI_4$			0.03			0.03	0.1	0.05	0.4	
$(2T)_2MASn_2I_7$			0.006		0.132	0.05	0.2	0.1	0.3	
$(3T)_2SnI_4$				0.005		0.03	0.2	0.1		0.5
$(3T)_2MASn_2I_7$				0.005	1.006	0.03	0.2	0.1		0.5

Table S2. Crystal data and structure refinement for $(BA)_2SnI_4$.

	$(BA)_2SnI_4$
Crystal data	
Chemical formula	C8 H24 I4 N2 Sn
M_r	774.58
Crystal system, space group	Orthorhombic, *Pbca*
Temperature (K)	150.00 K
a, *b*, *c* (Å)	8.4372(9), 8.9376(10), 26.167(3)
α, β, γ (°)	90, 90, 90
V ($Å^3$)	1973.2(4)
Z	4
F (000)	1392
D_x (Mg m^{-3})	2.607
Radiation type	Mo *K*α
No. of reflections for cell measurement	9239
θ range (°) for cell measurement	3.11 to 27.46°
μ (mm^{-1})	7.536
Crystal shape	Plate
Colour	Red
Crystal size (mm)	0.295 × 0.212 × 0.158
Data collection	
Diffractometer	Bruker AXS D8 Venture<br>diffractometer with Photon III charge-integrating pixel array detector (CPAD)
Radiation source	High-intensity diamond-Cu/Mo hybrid dual-microfocal X-ray light source
Monochromator	Triumph curved graphite crystal
Photon III charge-integrating pixel array detector	10 * 14 cm^2
Scan method	w and phi scans
Absorption correction	Multi-scan<br>*SADABS* 2016/2: Krause, L., Herbst-Irmer, R., Sheldrick G.M. & Stalke D. (2015). J. Appl. Cryst. 48, 3-10.
T_{min}, T_{max}	0.5755, 0.7456
No. of measured, independent and observed [$I > 2s(I)$] reflections	32126, 1512, 1456
R_{int}	0.0692
θ values (°)	$θ_{max}$ = 24.712, $θ_{min}$ = 2.873
$(\sin θ/λ)_{max}$ ($Å^{-1}$)	0.714

Range of *h*, *k*, *l*	-9<=h<=9, -10<=k<=10, -30<=l<=30
Refinement	
Refinement on	F^2
$R[F^2 > 2\sigma(F^2)]$, $wR(F^2)$, S	0.0810, 0.1634, 1.242
No. of reflections	1512
No. of parameters	127
No. of restraints	174
H-atom treatment	H-atom parameters constrained
Weighting scheme	$w = 1/[\sigma^2(F_o^2) +265.5513P]$ where $P = (F_o^2 + 2F_c^2)/3$
$(\Delta/\sigma)_{max}$	0.001
$\Delta\rho_{max}$, $\Delta\rho_{min}$ (e Å^{-3})	1.981 and -2.542

Description of $(BA)_2SnI_4$:

There has displace disorder for the Sn-I lattice. Uij components of ADPs of all C and N atoms (ISOR 0.001), SIMU 0.001 commands of Shelxl were used to maintain the similar anisotropic temperature factor. Because the crystal produces some diffraction points different from that of the single cell at low temperature, some diffraction points were deleted that do not meet the refined structure.

Table S3. Crystal data and structure refinement for $(BA)_2MASn_2I_7$.

	$(BA)_2MASn_2I_7$
Crystal data	
Chemical formula	C9 H30 I7 N3 Sn2
M_r	1306.04
Crystal system, space group	Orthorhombic, $Cmc2_1$
Temperature (K)	285.00 K
a, *b*, *c* (Å)	39.315(5), 8.8503(12), 8.7792(13)
α, β, γ (°)	90, 90, 90
V (Å^3)	3054.7(7)
Z	4
F (000)	2304
D_x (Mg m^{-3})	2.840
Radiation type	Mo $K\alpha$
No. of reflections for cell measurement	1498
θ range (°) for cell measurement	3.62 to 24.596°
μ (mm^{-1})	8.709
Crystal shape	Plate
Colour	black
Crystal size (mm)	0.233 × 0.091 × 0.091
Data collection	
Diffractometer	Bruker AXS D8 Venture<br>diffractometer with Photon III charge-integrating pixel array detector (CPAD)
Radiation source	High-intensity diamond-Cu/Mo hybrid dual-microfocal X-ray light source
Monochromator	Triumph curved graphite crystal
Photon III charge-integrating pixel array detector	10 * 14 cm^2
Scan method	w and phi scans
Absorption correction	Multi-scan<br>*SADABS* 2016/2: Krause, L., Herbst-Irmer, R., Sheldrick G.M. & Stalke D. (2015). J. Appl. Cryst. 48, 3-10.
T_{min}, T_{max}	0.6214, 0.7456
No. of measured, independent and observed [$I > 2s(I)$] reflections	7910, 2572, 1689
R_{int}	0.0835
θ values (°)	$\theta_{max} = 24.704$, $\theta_{min} = 2.359$
$(\sin \theta/\lambda)_{max}$ (Å^{-1})	0.714

Range of *h*, *k*, *l*	-46<=h<=42, -10<=k<=9, -10<=l<=10
Refinement	
Refinement on	F^2
$R[F^2 > 2\sigma(F^2)]$, $wR(F^2)$, S	0.0542, 0.1356, 1.050
No. of reflections	2572
No. of parameters	111
No. of restraints	81
H-atom treatment	H-atom parameters constrained
Weighting scheme	$w = 1/[\sigma^2(F_o^2) + (0.0281P)^2 + 21.7906P]$<br>where $P = (F_o^2 + 2F_c^2)/3$
$(\Delta/\sigma)_{max}$	0.001
$\Delta\rho_{max}$, $\Delta\rho_{min}$ (e Å^{-3})	0.741 and -0.922
Absolute structure parameter	0.2(3)

Description for $(BA)_2MASn_2I_7$:

The crystal structure has been refined as twin consist of inversion twinning in the structure. Conditions of the twin transformation matrix twin law (-1.0, 0.0, 0.0, 0.0, -1.0, 0.0, 0.0, 0.0, -1.0) combined with inversion twinning resulted in BASF values of 0.2(3).

Similar to Sn-I inorganic fragment of $(BA)_2SnI_4$, there has no disorder for the Sn-I lattice. All the BA cations were restrained to have similar and rational geometries according to the previous rationally BA cations model. ADPs and of disordered C, N atoms were restrained to be similar by the ISOR 0.001 and DELU 0.001 command of Shelxl. The similar anisotropic temperature factor and Uij components of C, N were restrained by SIMU 0.001 command of Shelxl. The distances of C-N, C-C have been restrained to meet rationally organic fragment by using the DFIX 0.01 of Shelxl command. According to the previous C-N, C-C distance of generally BA cations, the specific commands are as follows: the C-N bond length is restrained as 1.5Å, the non-π-conjugated C-C bond length is restrained as 1.5Å. All the disordered moieties of each cation were each restrained to have similar geometries based on the previous rationally BA cations model.

Table S4. Crystal data and structure refinement for $(BA)_2MA_2Sn_3I_{10}$.

	$(BA)_2MA_2Sn_3I_{10}$
Crystal data	
Chemical formula	C10 H36 I10 N4 Sn3
M_r	1837.81
Crystal system, space group	Orthorhombic, *Aea*2
Temperature (K)	274.00 K

a, *b*, *c* (Å)	8.8507(6), 51.957(4), 8.7981(5)
α, β, γ (°)	90, 90, 90
V (Å^3)	4045.8(5)
Z	4
F (000)	3217
D_x (Mg m^{-3})	3.017
Radiation type	Mo $K\alpha$
No. of reflections for cell measurement	1292
θ range (°) for cell measurement	3.470 to 24.547
μ (mm^{-1})	9.478
Crystal shape	Plate
Colour	Black
Crystal size (mm)	0.21 × 0.123× 0.123
Data collection	
Diffractometer	Bruker AXS D8 Venture<br>diffractometer with Photon III charge-integrating pixel array detector (CPAD)
Radiation source	High-intensity diamond-Cu/Mo hybrid dual-microfocal X-ray light source
Monochromator	Triumph curved graphite crystal
Photon III charge-integrating pixel array detector	10 * 14 cm^2
Scan method	w and phi scans
Absorption correction	Multi-scan<br>*SADABS* 2016/2: Krause, L., Herbst-Irmer, R., Sheldrick G.M. & Stalke D. (2015). J. Appl. Cryst. 48, 3-10.
T_{min}, T_{max}	0.6312, 0.7456
No. of measured, independent and observed [$I > 2s(I)$] reflections	13353, 4503, 2465
R_{int}	0.0748
θ values (°)	2.431 to 27.502
$(\sin \theta/\lambda)_{max}$ (Å^{-1})	0.714
Range of *h*, *k*, *l*	-10<=h<=10, -67<=k<=60, -11<=l<=11
Refinement	
Refinement on	F^2
$R[F^2 > 2\sigma(F^2)]$, $wR(F^2)$, S	0.0567, 0.1161, 1.037
No. of reflections	4503
No. of parameters	150

No. of restraints	73
H-atom treatment	H-atom parameters constrained
Weighting scheme	$w = 1/[\sigma^2(F_o^2) + (0.0363P)^2 + 60.992P]$<br>where $P = (F_o^2 + 2F_c^2)/3$
$(\Delta/\sigma)_{max}$	0.001
$\Delta\rho_{max}$, $\Delta\rho_{min}$ (e Å^{-3})	0.828 and -1.230
Absolute structure parameter	0.8(2)

Description for $(BA)_2MA_2Sn_3I_{10}$:

The crystal structure has been refined as twin consist of inversion twinning in the structure. Conditions of the twin transformation matrix twin law (-1.0, 0.0, 0.0, 0.0, -1.0, 0.0, 0.0, 0.0, -1.0), combined with inversion twinning resulted in BASF values of 0.8(3).

Different from Sn-I inorganic fragment of $(BA)_2SnI_4$ and $(BA)_2MASn_2I_7$, there has a small quantity of disorder for the Sn-I lattice. the I1 and I6 atoms was refined as disordered. all the occupancy of disordered I atoms were freely refined as I1 (0.8913) and I6 (0.1114). All the BA cations were restrained to have similar and rational geometries according to the previous rationally BA cations model. ADPs and of disordered C, N atoms were restrained to be similar by the ISOR 0.001 and DELU 0.001 command of Shelxl. The similar anisotropic temperature factor and Uij components of C, N were restrained by SIMU 0.001 command of Shelxl. The distances of C-N, C-C have been restrained to meet rationally organic fragment by using the DFIX 0.01 of Shelxl command. According to the previous C-N, C-C distance of generally BA cations, the specific commands are as follows: the C-N bond length is restrained as 1.5Å, the non-π-conjugated C-C bond length is restrained as 1.5Å. All the disordered moieties of each cation were each restrained to have similar geometries based on the previous rationally BA cations model.

Table S5. Crystal data and structure refinement for $(BA)_2MA_3Sn_4I_{13}$.

	$(BA)_2MA_3Sn_4I_{13}$
Crystal data	
Chemical formula	C11 H42 I13 N5 Sn4
M_r	2368.64
Crystal system, space group	Orthorhombic, $Cmc2_1$
Temperature (K)	301.00 K
a, b, c (Å)	64.447(4), 8.8422(6), 8.8027(6)
α, β, γ (°)	90, 90, 90
V (Å^3)	5016.2(6)
Z	4
F (000)	4127

D_x (Mg m^{-3})	3.136
Radiation type	Mo $K\alpha$
No. of reflections for cell measurement	1225
θ range (°) for cell measurement	3.401 to 24.433
μ (mm^{-1})	9.980
Crystal shape	Plate
Colour	Black
Crystal size (mm)	0.265 × 0.161× 0.161
Data collection	
Diffractometer	Bruker AXS D8 Venture<br>diffractometer with Photon III charge-integrating pixel array detector (CPAD)
Radiation source	High-intensity diamond-Cu/Mo hybrid dual-microfocal X-ray light source
Monochromator	Triumph curved graphite crystal
Photon III charge-integrating pixel array detector	10 * 14 cm^2
Scan method	w and phi scans
Absorption correction	Multi-scan<br>*SADABS* 2016/2: Krause, L., Herbst-Irmer, R., Sheldrick G.M. & Stalke D. (2015). J. Appl. Cryst. 48, 3-10.
T_{min}, T_{max}	0.6026, 0.7456
No. of measured, independent and observed [$I > 2s(I)$] reflections	16794, 5536, 2683
R_{int}	0.0837
θ values (°)	2.325 to 27.465
$(\sin \theta/\lambda)_{max}$ (Å^{-1})	0.714
Range of *h*, *k*, *l*	-67<=h<=83, -11<=k<=11, -10<=l<=10
Refinement	
Refinement on	F^2
$R[F^2 > 2\sigma(F^2)]$, $wR(F^2)$, S	0.0581, 0.1176, 1.049
No. of reflections	5536
No. of parameters	176
No. of restraints	98
H-atom treatment	H-atom parameters constrained
Weighting scheme	$w = 1/[\sigma^2(F_o^2) + (0.0350P)^2 + 49.1520P]$<br>where $P = (F_o^2 + 2F_c^2)/3$
$(\Delta/\sigma)_{max}$	0.001
$\Delta\rho_{max}$, $\Delta\rho_{min}$ (e Å^{-3})	1.178 and -1.459

Absolute structure parameter	0.1(3)

Description for $(BA)_2MA_3Sn_4I_{13}$:

The crystal structure has been refined as twin consist of inversion twinning in the structure. Conditions of the twin transformation matrix twin law (-1.0, 0.0, 0.0, 0.0, -1.0, 0.0, 0.0, 0.0, -1.0) combined with inversion twinning resulted in BASF values of 0.1(3).

Similar to Sn-I inorganic fragment of $(BA)_2MA_2Sn_3I_{10}$, there has a small quantity of disorder for the Sn-I lattice. the I6 and I8 atoms was refined as disordered. all the occupancy of disordered I atoms were freely refined as I6 (0.08731 and I8 (0.0.91088). Actually, the BA and MA cations were restrained to have similar and rational geometries according to the previous rationally model. ADPs and of disordered C, N atoms were restrained to be similar by the ISOR 0.001 and DELU 0.001 command of Shelxl. The similar anisotropic temperature factor and Uij components of C, N were restrained by SIMU 0.001 command of Shelxl. The distances of C-N, C-C have been restrained to meet rationally organic fragment by using the DFIX 0.01 of Shelxl command. According to the previous C-N, C-C distance of generally BA cations, the specific commands are as follows: the C-N bond length is restrained as 1.5Å, the non-π-conjugated C-C bond length is restrained as 1.5Å. All the disordered moieties of each cation were each restrained to have similar geometries based on the previous rationally model.

Table S6. Fitting parameters of exciton-photon coupling and Arrhenius extracted binding energies in different single crystals

	Γ_0 (meV)	Γ_{LO} (meV)	E_{LO} (meV)	Arrhenius extracted exciton binding energies (meV)
$(BA)_2SnI_4$	21±1	547±150	53±5	93±10
$(BA)_2MASn_2I_7$	48±1	228±53	48±5	49±3
$(BA)_2MA_2Sn_3I_{10}$	52±1	136±38	38±5	39±2
$(BA)_2MA_3Sn_4I_{13}$	22±1	62±12	19±3	35±4
$(PEA)_2SnI_4$	16±1	405±64	42±3	49±2
$(2T)_2SnI_4$	25±0.5	434±49	51±2	158±19
$(3T)_2SnI_4$	25±3	354±158	43±8	52±5
$(PEA)_2MASn_2I_7$	13±2	198±29	28±3	40±3
$(2T)_2MASn_2I_7$	43±1	190±52	43±6	69±5
$(3T)_2MASn_2I_7$	44±2	142±30	30±4	34±10

Table S7. Crystal data and structure refinement for $(PEA)_2SnI_4$.

	$(PEA)_2SnI_4$
Crystal data	
Chemical formula	C16 H24 I4 N2 Sn
M_r	C16 H24 I4 N2 Sn
Crystal system, space group	Triclinic, *P*-1
Temperature (K)	150.00 K
a, *b*, *c* (Å)	8.6441(7), 8.6529(7), 16.4510(14)
α, β, γ (°)	94.678(3), 100.367(3), 90.489(3)
V ($Å^3$)	1206.02(17)
Z	2
F (000)	792
D_x (Mg m^{-3})	2.398
Radiation type	Mo *K*α
No. of reflections for cell measurement	9956
θ range (°) for cell measurement	2.526 to 27.572
μ (mm^{-1})	6.180
Crystal shape	Plate
Colour	Red
Crystal size (mm)	0.200× 0.210× 0.160
Data collection	
Diffractometer	Bruker AXS D8 Venture<br>diffractometer with Photon III charge-integrating pixel array detector (CPAD)
Radiation source	High-intensity diamond-Cu/Mo hybrid dual-microfocal X-ray light source
Monochromator	Triumph curved graphite crystal
Photon III charge-integrating pixel array detector	10 * 14 cm^2
Scan method	w and phi scans
Absorption correction	Multi-scan<br>*SADABS* 2016/2: Krause, L., Herbst-Irmer, R., Sheldrick G.M. & Stalke D. (2015). J. Appl. Cryst. 48, 3-10.
T_{min}, T_{max}	0.6090, 0.7456
No. of measured, independent and observed [$I > 2s(I)$] reflections	24101, 4083, 3798
R_{int}	0.0407

θ values (°)	2.396 to 24.713
$(\sin \theta/\lambda)_{max}$ (Å^{-1})	0.714
Range of *h*, *k*, *l*	-10<=h<=10, -10<=k<=10, -19<=l<=19
Refinement	
Refinement on	F^2
$R[F^2 > 2\sigma(F^2)]$, $wR(F^2)$, S	0.0367, 0.0831, 1.223
No. of reflections	4084
No. of parameters	334
No. of restraints	216
H-atom treatment	H-atom parameters constrained
Weighting scheme	$w = 1/[\sigma^2(F_o^2) +16.1177P]$<br>where $P = (F_o^2 + 2F_c^2)/3$
$(\Delta/\sigma)_{max}$	0.001
$\Delta\rho_{max}$, $\Delta\rho_{min}$ (e Å^{-3})	0.971 and -1.113

Description for $(PEA)_2SnI_4$ crystal:

After analyzing the overall inorganic and organic framework, I3 and I5 atoms approximately located in the pseudo-translated positions, there has two large residual electron peaks around I2 an I6. the I atoms of the Sn-I lattice were refined as a small amount of disordered along the ab plane, the iodide atoms along the [001] direction have no disordered. The distance between large residual electron peaks and Sn was closed to Sn-I bond length. According to the residual electron peak 9.6 e Å^{-3}, it was defined as I atoms using the free refine method, the free refined I atoms has 0.086 and 0.075 occupancy. In order to maintain the electrically neutral, the occupancy of I5 and I3 atoms were modified as 0.07 and 0.93 respectively, ADPs of disordered I5 and I3 atoms were restrained to be similar (ISOR 0.001 0.002 I5 I3).

Since a small amount I atoms are located in a pseudosymmetric position, resulting in the substantial disorder of organic cations and substantial correlation position for ADPs of C atoms. To control correlation all organic phenethylammonium cations were restrained to have rational and similar geometries. Uij components of ADPs of all C9 and C10 atoms (ISOR 0.01 0.02 C9 C10), SIMU 0.01 commands of Shelxl were used to maintain the similar anisotropic temperature factor. There is some deviation in the position of the terminal C and N atom, the distance of C2 and C4, C9 and C10, N2 and C10 have been rationally restricted (DFIX 2.5 0.01 C2 C4 DFIX 1.5 0.01 C9 C10 DFIX 2.5 0.01 N2 C10).

Table S8. Crystal data and structure refinement for $(PEA)_2MASn_2I_7$.

	$(PEA)_2MASn_2I_7$
Crystal data	
Chemical formula	C17 H30 I7 N3 Sn2
M_r	1402.12
Crystal system, space group	Triclinic, *P*-1
Temperature (K)	150.00 K
a, *b*, *c* (Å)	8.6871(5), 8.6913(6), 22.4509(15)
α, β, γ (°)	97.479(2), 93.615(2), 90.265(2)
V (Å^3)	1677.19(19)
Z	2
F (000)	1248
D_x (Mg m^{-3})	2.776
Radiation type	Mo *K*α
No. of reflections for cell measurement	9819
θ range (°) for cell measurement	2.36 to 27.50
μ (mm^{-1})	7.942
Crystal shape	Plate
Colour	Black
Crystal size (mm)	0.155 × 0.123 × 0.05
Data collection	
Diffractometer	Bruker AXS D8 Venture<br>diffractometer with Photon III charge-integrating pixel array detector (CPAD)
Radiation source	High-intensity diamond-Cu/Mo hybrid dual-microfocal X-ray light source
Monochromator	Triumph curved graphite crystal
Photon III charge-integrating pixel array detector	10 * 14 cm^2
Scan method	w and phi scans
Absorption correction	Multi-scan<br>*SADABS* 2016/2: Krause, L., Herbst-Irmer, R., Sheldrick G.M. & Stalke D. (2015). J. Appl. Cryst. 48, 3-10.
T_{min}, T_{max}	0.4103, 0.7455
No. of measured, independent and observed [$I > 2s(I)$] reflections	37607, 5528, 5451
R_{int}	0.0304
θ values (°)	2.349 to 24.713
$(\sin \theta/\lambda)_{max}$ (Å^{-1})	0.714

Range of *h*, *k*, *l*	*h* = -11 - 11, *k* = -11 - 11, *l* = -26 - 26
Refinement	
Refinement on	F^2
$R[F^2 > 2\sigma(F^2)]$, $wR(F^2)$, *S*	0.029, 0.0671, 1.232
No. of reflections	5528
No. of parameters	389
No. of restraints	595
H-atom treatment	H-atom parameters constrained
Weighting scheme	$w = 1/[\sigma^2(F_o^2) + 13.644P]$<br>where $P = (F_o^2 + 2F_c^2)/3$
$(\Delta/\sigma)_{max}$	0.001
$\Delta\rho_{max}$, $\Delta\rho_{min}$ (e Å^{-3})	1.769 and -1.099

Description for $(PEA)_2MASn_2I_7$:

There has no disorder for the Sn-I lattice. But the organic part, some C, N atoms display the unusual temperature factors and distances. In order to control all organic phenethylammonium cations were restrained to have rational and similar geometries. Uij components of ADPs of all C and N atoms (ISOR 0.01), SIMU 0.01 commands of Shelxl were used to maintain the similar anisotropic temperature factor. There is some deviation in the position of the terminal C and N atom, their distances have been rationally restricted (DFIX 1.55 0.01 N4 C11, DFIX 1.55 0.01 C11 C12, DFIX 1.55 0.01 N1 C1, DFIX 1.55 0.01 C4 C5, DFIX 1.55 0.01 C12 C13, DFIX 2.35 0.01 N4 C12, DFIX 1.55 0.01 C2 N2).

Table S9. Crystal data and structure refinement for $(2T)_2SnI_4$.

	$(2T)_2SnI_4$
Crystal data	
Chemical formula	C20 H24 I4 N2 S4 Sn
M_r	1046.94
Crystal system, space group	Monoclinic, *Cm*
Temperature (K)	150.00
a, *b*, *c* (Å)	8.5791(8), 40.673(6), 6.0303(8)
α, β, γ (°)	90, 134.654(6), 90
V (Å^3)	1496.8(3)
Z	2
F (000)	968
D_x (Mg m^{-3})	2.323
Radiation type	Mo *K*α
No. of reflections for cell measurement	8553
θ range (°) for cell measurement	3.005 to 26.615
μ (mm^{-1})	5.270
Crystal shape	Plate
Colour	Red
Crystal size (mm)	0.221 × 0.125× 0.05
Data collection	
Diffractometer	Bruker AXS D8 Venture<br>diffractometer with Photon III charge-integrating pixel array detector (CPAD)
Radiation source	High-intensity diamond-Cu/Mo hybrid dual-microfocal X-ray light source
Monochromator	Triumph curved graphite crystal
Photon III charge-integrating pixel array detector	10 * 14 cm^2
Scan method	w and phi scans
Absorption correction	Multi-scan<br>*SADABS* 2016/2: Krause, L., Herbst-Irmer, R., Sheldrick G.M. & Stalke D. (2015). J. Appl. Cryst. 48, 3-10.
T_{min}, T_{max}	0.6304, 0.7456
No. of measured, independent and observed [$I > 2s(I)$] reflections	21431, 3249, 2638
R_{int}	0.0762
θ values (°)	3.376 to 27.460
(sin θ/λ)$_{max}$ (Å^{-1})	0.714

Range of *h*, *k*, *l*	-11<=h<=11, -52<=k<=52, -7<=l<=7
Refinement	
Refinement on	F^2
$R[F^2 > 2\sigma(F^2)]$, $wR(F^2)$, S	0.0626, 0.1565, 1.077
No. of reflections	3249
No. of parameters	250
No. of restraints	394
H-atom treatment	H-atom parameters constrained
Weighting scheme	$w = 1/[\sigma^2(F_o^2) + (0.0771P)^2 + 32.6155P]$<br>where $P = (F_o^2 + 2F_c^2)/3$
$(\Delta/\sigma)_{max}$	0.001
$\Delta\rho_{max}$, $\Delta\rho_{min}$ (e Å^{-3})	2.953 and -1.404
Absolute structure	0.37(2)

Description For $(2T)_2SnI_4$:

There has been refined as inversion twinning in the structure. Conditions of the twin transformation matrix twin law (-1.0, 0.0, 0.0, 0.0, -1.0, 0.0, 0.0, 0.0, -1.0) combined with inversion twinning resulted in BASF values of 0.4(3).

The Sn-I part of the lattice was refined as disordered. The disordered Sn and I atoms have four-fold symmetry or higher symmetry for the Sn-I lattice if occupancy rate of every disordered part would be equal. ADPs of disordered Sn-I atoms were restrained to be similar (ISOR 0.001, DELU 0.001 command of Shelxl). All the occupancy of disordered Sn-I layer were refined as 0.5 occupancy due to the mirror symmetry of this space group. These Sn and I atoms in special positions are refined as half occupied including I1 (0.5), I2 (0.5), I3 (0.5), I4 (0.5), Sn1 (0.5).

Because the observed disorder in the Sn-I lattices, it creates disorder for the ammonium moieties of the 2T cations. All the 2T cations were restrained to have similar and rational geometries according to the previous rationally 2T cations model. ADPs and of disordered C, N, S atoms were restrained to be similar by the ISOR 0.001 command of Shelxl. The similar anisotropic temperature factor and Uij components of C, N, S were restrained by SIMU 0.001 command of Shelxl. Because of the π-conjugated in the rigid 2T cations, the thiophene units were restrained to be flat. The distances of C-N, C-S, C-C have been restrained to meet rationally organic fragment by using the DFIX 0.01 of Shelxl command. According to the previous C-N, C-S, C-C distance of generally 2T cations, the specific commands are as follows: the C-N bond length is restrained as 1.55 Å, the π-conjugated C-C bond length is restrained as 1.4 Å, the non-π-conjugated C-C bond length is restrained as 1.55 Å, the C-S bond length is restrained as 1.65 Å. All the disordered moieties of each cation were each restrained to have similar geometries based on the previous rationally 2T cations model.

Table S10. Crystal data and structure refinement for $(3T)_2SnI_4$.

	$(3T)_2SnI_4$
Crystal data	
Chemical formula	C28 H28 I4 N2 S6 Sn
M_r	1211.17
Crystal system, space group	Monoclinic, *PC*
Temperature (K)	150.00
a, *b*, *c* (Å)	24.904(2), 12.0434(11), 12.2044(11)
α, β, γ (°)	90, 97.054(3), 90
V ($Å^3$)	3632.7(6)
Z	4
F (000)	2272
D_x (Mg m^{-3})	2.215
Radiation type	Mo *K*α
No. of reflections for cell measurement	9813
θ range (°) for cell measurement	2.47 to 27.48
μ (mm^{-1})	4.471
Crystal shape	Plate
Colour	Red
Crystal size (mm)	0.321× 0.125 × 0.05
Data collection	
Diffractometer	Bruker AXS D8 Venture<br>diffractometer with Photon III charge-integrating pixel array detector (CPAD)
Radiation source	High-intensity diamond-Cu/Mo hybrid dual-microfocal X-ray light source
Monochromator	Triumph curved graphite crystal
Photon III charge-integrating pixel array detector	10 * 14 cm^2
Scan method	w and phi scans
Absorption correction	Multi-scan<br>*SADABS* 2016/2: Krause, L., Herbst-Irmer, R., Sheldrick G.M. & Stalke D. (2015). J. Appl. Cryst. 48, 3-10.
T_{min}, T_{max}	0.6308, 0.7456
No. of measured, independent and observed [$I > 2s(I)$] reflections	94022, 12090, 9483
R_{int}	0.0663
θ values (°)	2.361 to 24.713

$(\sin \theta/\lambda)_{max}$ (Å^{-1})	0.714
Range of *h*, *k*, *l*	-10<=h<=10, -10<=k<=10, -29<=l<=29
Refinement	
Refinement on	F^2
$R[F^2 > 2\sigma(F^2)]$, $wR(F^2)$, S	0.0563, 0.1645, 1.07
No. of reflections	12090
No. of parameters	780
No. of restraints	998
H-atom treatment	H-atom parameters constrained
Weighting scheme	$w = 1/[\sigma^2(F_o^2) + (0.0996P)^2 + 6.2583P]$ where $P = (F_o^2 + 2F_c^2)/3$
$(\Delta/\sigma)_{max}$	0.001
$\Delta\rho_{max}$, $\Delta\rho_{min}$ (e Å^{-3})	2.454 and -2.111
Absolute structure	0.16(18)

Description For $(3T)_2SnI_4$:

The crystal structure has been refined as twin consist of pseudo-merohedral twinning (emulating monoclinic symmetry) and inversion twinning in the structure. Conditions of the twin transformation matrix twin law (-1.0, 0.0, 0.0, 0.0, -1.0, 0.0, 0.0, 0.0, -1.0) combined with inversion twinning resulted in BASF values of 0.84(18).

The structure exhibits pseudo-translation along the [010] direction. The [010] translational symmetry has been broken by modulation of the Sn-I layer and by the positions of the terminal part of 3T organic groups.

Similar to the $(2T)_2SnI_4$ crystal structure, the Sn-I part of the lattice was refined as disordered. The disordered Sn and I atoms have four-fold symmetry or higher symmetry for the Sn-I lattice if occupancy rate of every disordered part would be equal. ADPs of disordered Sn-I atoms were restrained to be similar (ISOR 0.001, DELU 0.001 command of Shelxl). Firstly, all the occupancy of disordered I atoms were freely refined as I6 (0.09809), I4 (0.06789), I8 (0.06485). In order to maintain electrically neutral, the occupancy of major and minor moiety I has been restrained as I3 (0.88), I5 (0.95), I7 (0.9) and I4 (0.12), I6 (0.05), I8 (0.1).

Because the observed disorder in the Sn-I lattices, it creates disorder for the ammonium moieties of the 3T cations along [010] direction. All the 3T cations were restrained to have similar and rational geometries according to the previous rationally 3T cations model. ADPs and of disordered C, N, S atoms were restrained to be similar by the ISOR 0.001 and DELU 0.001 command of Shelxl. The similar anisotropic temperature factor and Uij components of C, N, S were restrained by SIMU 0.001 command of Shelxl. Because of the π-conjugated in the rigid 3T cations, the thiophene units were restrained to be flat. The distances of C-N, C-S, C-C have been restrained to meet rationally organic fragment by using the DFIX 0.01 of Shelxl command. According to the previous C-N, C-S, C-C distance of generally 3T cations, the specific commands are as follows: the C-N bond

length is restrained as 1.55 Å, the π-conjugated C-C bond length is restrained as 1.4 Å, the non-π-conjugated C-C bond length is restrained as 1.55 Å, the C-S bond length is restrained as 1.65 Å. All the disordered moieties of each cation were each restrained to have similar geometries based on the previous rationally 3T cations model.
